# Composition, Stratigraphy, and Geological History of the Noachian Basement Surrounding the Isidis Impact Basin

**DOI:** 10.1029/2019je006190

**Published:** 2020-03-10

**Authors:** Eva L. Scheller, Bethany L. Ehlmann

**Affiliations:** 1 Division of Geological and Planetary Sciences, California Institute of Technology, Pasadena, CA, USA,; 2 Jet Propulsion Laboratory, California Institute of Technology, Pasadena, CA, USA

## Abstract

The western part of the Isidis basin structure hosts a well-characterized Early Noachian to Amazonian stratigraphy. The Noachian Basement comprises its oldest exposed rocks (Early to Mid-Noachian) and was previously considered a single low-Ca pyroxenes (LCP)- and Fe/Mg-smectite-bearing unit. Here, we divide the Noachian Basement Group into five distinct geological units (Stratified Basement Unit, Blue Fractured Unit, Mixed Lithology Plains Unit, LCP-bearing Plateaus Unit, and Fe/Mg-smectite-bearing Mounds Unit), two geomorphological features (megabreccia and ridges), and a mineral deposit (kaolinite-bearing bright materials), based on geomorphology, spectral characteristics, and stratigraphic relationships. Megabreccia contain four different pre-Isidis lithologies, possibly including deeper crust or mantle materials, formed through mass wasting associated with transient crater collapse during Isidis basin formation. The Fe/Mg-smectite-bearing Stratified Basement Unit and LCP-bearing Blue Fractured Unit likewise represent pre-Isidis units within the Noachian Basement Group. Multiple Fe/Mg-smectite-bearing geological units with different stratigraphic positions and younger kaolinite-bearing bright materials indicate several aqueous alteration episodes of different ages and styles. Units with slight changes in pyroxene spectral properties suggest a transition from low-Ca pyroxene-containing materials to those with higher proportions of pyroxenes higher in Ca and/or glass that could be related to different impact and/or igneous processes, or provenance. This long history of Noachian and potentially Pre-Noachian geological processes, including impact basin formation, aqueous alteration, and multiple igneous and sedimentary petrogeneses, records changing ancient Mars environmental conditions. All units defined by this study are available 20 km outside of Jezero crater for in situ analysis and sampling during a potential extended mission scenario for the Mars 2020 rover.

## Plain Language Summary

The Isidis basin’s Noachian Basement Group is a collection of geological materials that are among the oldest rocks exposed on the Martian surface (>3.8 billion years old). We have characterized their spectral signatures and appearances using instruments on the Mars Reconnaissance Orbiter. The Noachian Basement Group contains eight different geological categories of rocks formed at different times and by different processes. One of these categories is megabreccia, which are blocks of rocks created when a meteorite impact formed the 1,900-km Isidis impact basin. The megabreccia contain materials from deep within Mars and likely formed during enormous landslides after the impact. The spectral properties of the megabreccia reveal that they contain four different types of rock that must have originated before the Isidis impact basin formed. Through investigations of the three-dimensional relationships between our eight categories of geological materials, we observe that they record multiple distinct water-rich environments, multiple igneous processes, and giant impact processes, during an ancient time period that we know little about. Furthermore, all eight different rock categories can be studied and sampled with the next Mars rover, set to launch in 2020, which will answer outstanding questions about the climate, astrobiology, and geological processes on early Mars.

## 1. Introduction

**Figure 1.**
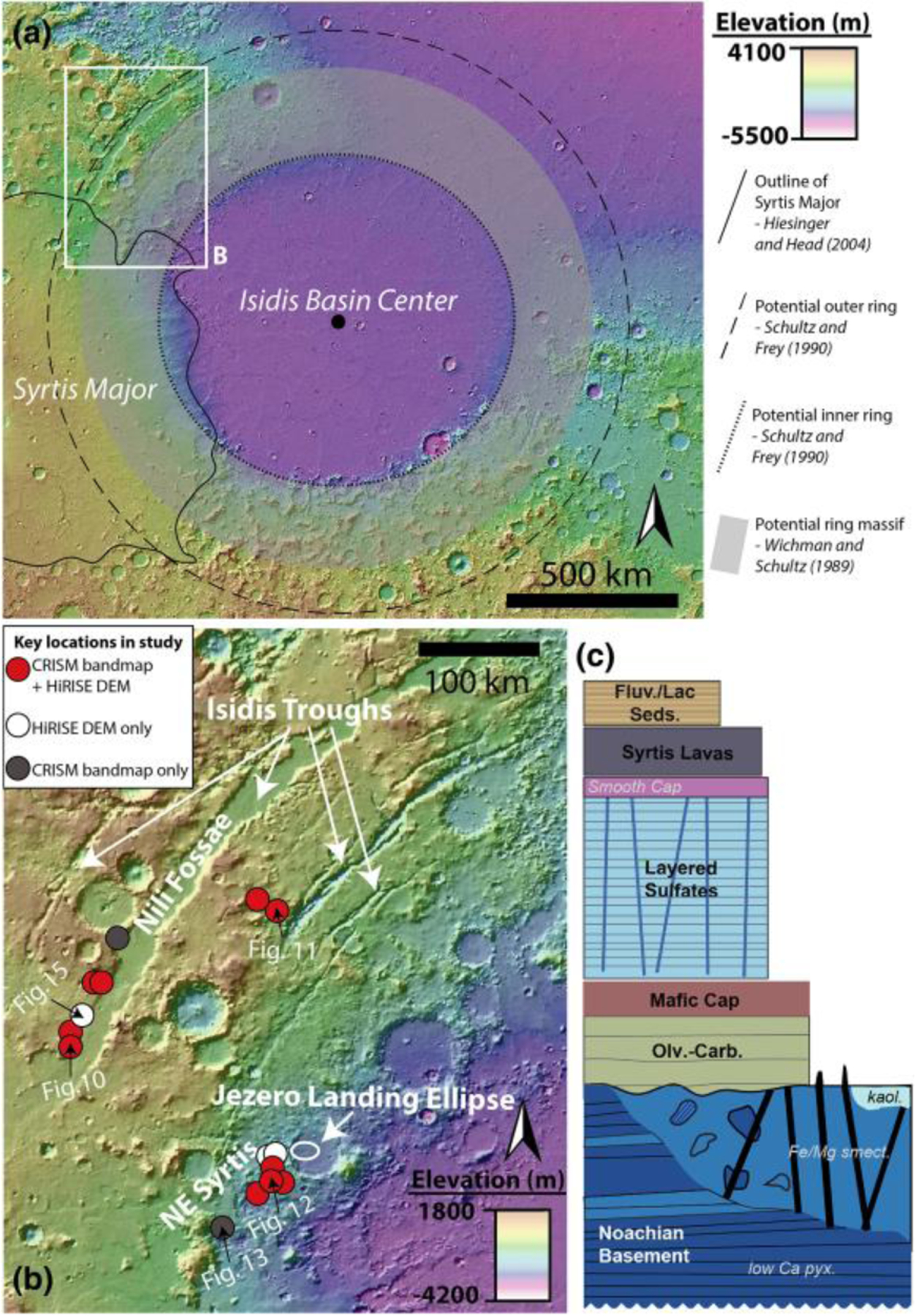
(a) MOLA topography map of Isidis basin and Syrtis Major. Outlines refer to suggested impact basin features from previous literature. White box denotes position of panel (b). (b) Map of main study area. Circles refer to key stratigraphic locations used in this study. Red circles refer to the position of CRISM bandmaps with HiRISE DEMs, white circles refer to HiRISE DEMs only, and the gray circle refers to CRISM bandmap only. The location of data shown in Figures 10–15 is indicated with black arrows. (c) Regional stratigraphy of study area within panel (b). The regional stratigraphy represents a summarization of Mustard et al. (2009), Ehlmann and Mustard (2012), Goudge et al. (2015), Bramble et al. (2017), and Quinn and Ehlmann (2019a).

Understanding the geological history of the ancient Pre-Noachian to Mid-Noachian crust on Mars is imperative as it includes processes such as impact basin formation, igneous petrogenesis, climate evolution, and ancient aqueous environments that are essential for understanding the origin, early evolution, and habitability of terrestrial planets. This time period encompasses rocks formed >3.82 Ga (Mandon et al., 2019; Werner & Tanaka, 2011). However, few well-exposed and well-preserved examples of Pre-Noachian to Noachian-aged crust exist on Mars and other solar system bodies. The NW region of the Isidis basin, a 1,900-km, 3.96–3.97 Ga (Fassett & Head, 2011; Werner, 2008), Early-Mid Noachian impact basin structure on the crustal dichotomy boundary (Ritzer & Hauck, 2009), provides a window into the geological history of ancient Mars that is exceptionally well preserved compared to rocks of the same age on Mars and Earth (Figure 1).

The NW Isidis basin region includes Nili Fossae, NE Syrtis, and the Jezero crater watershed and contains a well-characterized Noachian to Amazonian stratigraphy (Figure 1) (Bramble et al., 2017; Ehlmann et al., 2009; Ehlmann & Mustard, 2012; Goudge et al., 2015; Mangold et al., 2007; Mustard et al., 2007; Mustard et al., 2009; Quinn & Ehlmann, 2019a). The lowermost part of this stratigraphy is the >~600-m-thick Noachian Basement Group (Bramble et al., 2017; Ehlmann & Mustard, 2012; Goudge et al., 2015; Mangold et al., 2007; Mustard et al., 2009). Regionally, the Noachian Basement Group is overlain by the olivine-carbonate-bearing fractured unit, various high-Ca pyroxene-bearing materials often referred to as the mafic cap unit, a sedimentary unit of layered sulfates, and Hesperian-age (Hiesinger, 2004) Syrtis Major lava flows. Previous studies using infrared remote sensing have determined that the Noachian Basement contains low-Ca pyroxenes (LCP), Fe/Mg-smectite, and kaolinite (Ehlmann et al., 2009; Ehlmann & Mustard, 2012; Mangold et al., 2007; Michalski et al., 2010; Mustard et al., 2009). Additionally, the Noachian Basement includes a variety of geomorphological features such as ridges (Pascuzzo et al., 2019; Saper & Mustard, 2013), smooth plains, knobby plains, mounds, and megabreccia (Bramble et al., 2017).

In addition to recording ancient aqueous environments and igneous petrogenesis, the Noachian Basement Group also records processes forming the Isidis basin. In particular, its megabreccia have been proposed to have formed by the Isidis impact (Mustard et al., 2009). However, previous literature has neither considered the exact formation mechanism of megabreccia nor the location of other Isidis impact products such as melt sheet and ejecta. Currently, the formation processes of multiring impact basins are not well understood as they are primarily based on models with few opportunities for constraints through field studies. Hydrocode and other modeling efforts have been performed primarily for lunar impact basins (Johnson et al., 2016; Schultz & Crawford, 2016) and the Chixulub impact basin (Baker et al., 2016; Collins et al., 2002). Study of lunar impact basins through satellite observations and sample analysis (Howard et al., 1974) and studies of the three largest impact basins on Earth (150- to 300-km diameters), including Vredefort (Reimold & Gibson, 1996), Sudbury (Riller, 2005) and the Chixulub drilling project (Morgan et al., 2016), have also contributed significantly to our understanding of impact basin formation processes. Hence, the Isidis Noachian Basement Group on Mars provides an extraordinary opportunity to further our understanding of these impact basin formation processes.

Although the diversity of the Noachian Basement Group has been evaluated in previous literature, the collective stratigraphic and geological histories of these various compositional and geomorphic units within the Noachian Basement Group have not been determined. In this study, we use combined mineralogical, geomorphological, and stratigraphic analyses in order to define units, their stratigraphic position, and, where possible, their formation history within the Noachian Basement Group. We intentionally adopt the nomenclature of “Group” to describe the basement because it formed during a time interval prior to formation of younger units in the regional stratigraphy but is clearly composed of multiple distinctive units with different ages and formation processes. Furthermore, we investigate the geographical distribution of the Isidis megabreccia, some of the oldest rocky materials exposed on solar system terrestrial planets, for the first time, and systematically classify the megabreccia lithologies. We test between the multiple megabreccia formation hypotheses (ballistic ejecta, melt flows, crater floor/peak fracturing, and gravitational flows), using the characteristics of distribution, texture, lithology, and block size of megabreccia that are expected to differ between formation mechanisms (Table 1). In turn, this provides constraints on the preservation (shock pressure, temperature, and strain) of the Pre-Noachian or Early Noachian materials within the megabreccia. We evaluate the potential presence of Isidis impact melt and ejecta in the new geological units defined in this study. Lastly, we provide a detailed map of the occurrence of these materials within potential driving distance of the Mars 2020 rover.

## 2. Methods

The composition and geomorphology of the Noachian Basement in the study area were analyzed using data from the Context Camera (Malin et al., 2007), High Resolution Imaging Science Experiment (HiRISE; McEwen et al., 2007), Mars Orbiter Laser Altimeter (MOLA; Zuber, 1992), Compact Reconnaissance Imaging Spectrometer for Mars (CRISM; Murchie et al., 2007), and Thermal Emission Imaging System (Christensen et al., 2004) data sets (Table 2), incorporated into an ArcGIS database. CRISM images were also analyzed in the ENVI software package.

### 2.1. Megabreccia Distribution Map

Within ~900 HiRISE images, we searched for large blocks, >1 m, within a radial distance of 500–2,000 km from the crater center, including the northwestern and south rim but excluding areas dominated by Syrtis Major and the Northern Plains (Figure 1a). In the Nili Fossae and NE Syrtis areas, we searched Noachian Basement units that were mapped by Bramble et al. (2017) and Goudge et al. (2015). Our mapping criteria for megabreccia were (1) occurrence within Noachian Basement and underlying the olivine-carbonate unit, (2) no association with ejecta blankets of other craters, (3) albedo contrast to surrounding matrix, (4) textural contrast to surrounding matrix, and (5) distinct blocky shape (typically angular or subrounded). Our mapping efforts are limited by the availability of HiRISE grayscale and color data (megabreccia are easier to observe in color data, Figure 3), exposure of the basement units (significantly better near grabens due to erosion), and dust/sand cover. Regions south and east within the Isidis structure have much less HiRISE coverage than the western part of the Isidis structure. In addition, much of the basement in these regions is observed in visible image data to be mantled by fine-grained materials, consistent with Thermal Emission Imaging System thermal inertia data (Bishop et al., 2013). HiRISE that we requested of Noachian regions near Libya Montes (as defined by Bishop et al., 2013) had a thick cover of fine-grained materials, obscuring any bedrock.

Outlining all individual clasts of all 173 megabreccia-bearing outcrops was beyond the scope of this study; however, we outlined all individual block clasts above HiRISE resolution within 13 outcrops (totaling 4,600 individual block clasts), representative of the 13 different distance and elevations bins from the Isidis crater center and tabulated their size characteristics (supporting information Data Set S2 and Figure 5). The largest outcrop(s) within each distance and elevation bin (Figure 5) were chosen for this outlining. The reported block size of these 4,600 individual block clasts represents a maximum length that was calculated by constructing a minimum enveloping circle to each megabreccia outline and calculating the diameter of this circle. In addition, we calculated the planar distance between the Isidis crater center and the center of the minimum enveloping circle of each megabreccia.

Megabreccia block sizes were then binned according to planar distance from Isidis crater center and MOLA elevation for construction of boxplots in order to investigate changes in megabreccia block sizes with crater distance and elevation. These binned block size data were examined via box plots created with the Python seaborn module (Waskom, 2017) in order to investigate any systematic changes in the median, quartiles, and ranges with distance or elevation. In addition, all of the block size distributions within distance and elevation bins were subjected to pairwise Mann-Whitney U test using the SciPy module (Virtanen et al., 2020) in order to test for any nonparametric differences between these distributions. Subsequently, the binned block size distributions were fitted with skewed normal distributions and lognormal distributions using the SciPy module in order to investigate any systematic changes to the mean, mode, variance, skewness, kurtosis, and overall shapes of distributions with distance and elevation.

### 2.2. Megabreccia Lithologies

**Figure 2.**
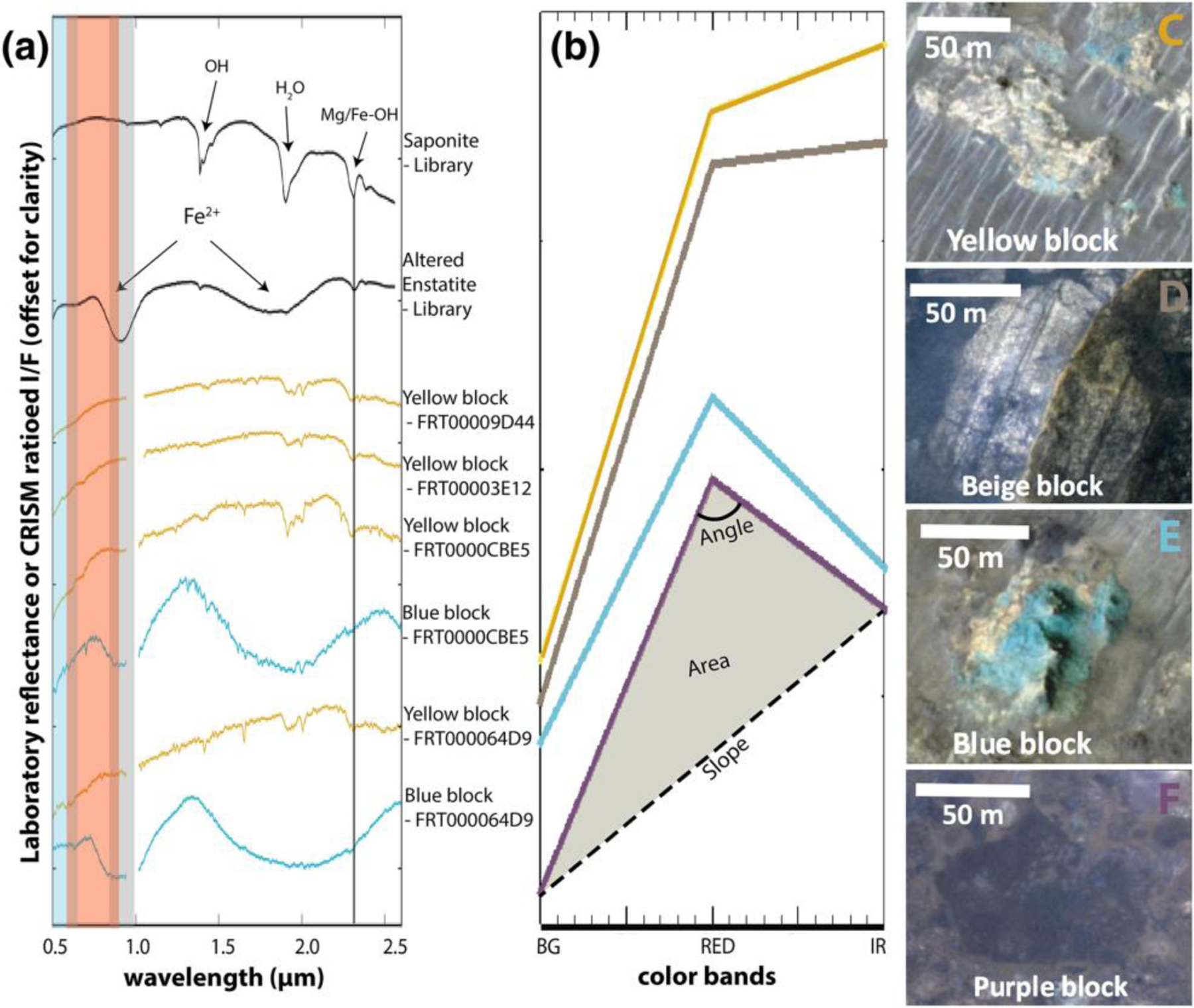
(a) CRISM spectra of six different megabreccia blocks from four different outcrops compared to two library spectra of saponite (Mg-smectite) and altered enstatite (LCP) from the USGS spectral library (Clark et al., 1993). Wavelength intervals colored blue, red, and gray correspond to the wavelength intervals of BG, RED, and IR bands of HiRISE color images. Center coordinate(s) of block(s) within CRISM image(s) FRT00009D44 is 20°2′10.15″N, 73°40′51.02″E, FRT00003E12 is 22°11′22.01″N, 77°4′24.28″E, FRT0000CBE5 are 17°17′34.46″N, 76°17′54.60″E and 17°17′33.32″N, 76°17′59.31″E, and FRT000064D9 are 21°6′5.22″N, 74°14′15.19″E and 21°6′26.69″N, 74°14′13.99″E. (b) Average HiRISE color band profiles of four different megabreccia blocks corresponding to blocks shown in (c)–(f). (c) and (e) are from HiRISE image ESP_047049_2015 (Outcrop ID 103 in Data Set S1), (d) is from ESP_033572_1995 (Outcrop ID 64 in Data Set S1, and (f) is from ESP_037185_2010 (Outcrop ID 50 in Data Set S1). These HiRISE color band profiles were parameterized through band ratios, slope (black stippled line), area (gray shaded area), and angle (solid black line).

CRISM covers 12 outcrops of megabreccia that were analyzed block by block through single-pixel study (Figure 2). As megabreccia blocks were generally below CRISM resolution, we also used HiRISE color data in order to analyze megabreccia. Following instructions by Eliason et al. (2007) and using GeospatialData Abstraction Library (GDAL), HiRISE color Digital Number (DN) were corrected to: $\frac{I}{F^{*}\text{cos}(\theta )}$, where *I* is the measured radiance, *F* is the radiance of the Sun, and *θ* is the Sun angle. All megabreccia within eight different HiRISE color images were outlined individually, and the three band values of the HiRISE color rasters were extracted for each pixel within this outline using arcpy tool Extract by Mask. For each pixel, we calculated band ratios of IR/RED, IR/BG, and BG/RED as suggested in Delamere et al. (2010). In addition, we calculated the slope, angle, and area of each HiRISE color band profile (Figure 2) through trigonometric formulas. Variations in these six parameters are typically due to absorptions associated with Fe^2+^ and Fe^3+^ in minerals (Figure 2). We created 2-D histograms of all pixel values for these six different parameters, identified megabreccia classes based on manually selected clusters, and then determined which visual colors from Figure 2 correlated with clusters and certain parameter values. Additionally, we analyzed four HiRISE color images that contained a variety of Noachian Basement Group units and the olivine-carbonate unit, defined using CRISM and HiRISE, using the same color parameterization.

Furthermore, we verified that results in HiRISE color parameter space reflect changes in lithology rather than changes in lighting and geometry. First, we analyzed 3 different HiRISE color images (ESP_047049_2015, ESP_045137_2015, and ESP_045071_2015) acquired over the same area at different times. The point clouds and 2-D histograms of these three images were compared visually. No significant differences were noted between these three different point clouds and 2-D histograms. Second, we compared HiRISE color results of the same megabreccia in direct sunlight and shadow. The area of profiles was affected slightly by shadow effects, although this difference is much smaller than the observed parameter differences between clusters in our 2-D histograms. Shadowed megabreccia appeared to have approximately the same values in all other parameters as megabreccia in sunlight.

Many megabreccia outcrops included multiple blocks of different color/albedo and textural properties. For blocks with HiRISE color coverage, the presence of blocks with visual color in the standard HiRISE IR-Red-BG product was recorded: blue/green (here called “blue”), yellow, beige, and purple. This color classification was done by eye for individual outcrops. Outcrops were classified based on whether they contained blocks of only a single color or multiple colors. We determined the textural properties by visual inspection: layered or not; uniformity or heterogeneity of albedos (labeled “monomict” and “polymict”), and proximity of adjacent blocks. Layered materials only exhibited albedo differences, not color differences, and could be classified with one color. We extracted longitude and latitude coordinates and elevation for each megabreccia outcrops from MOLA data (SimpleCylindrical_Mars map projection; see Data Set S1).

### 2.3. Defining Geological Units

Approximately 30 CRISM TRR3 images covering the western rim of the Isidis basin structure were analyzed in order to define spectral characteristics of the Noachian Basement. These 30 CRISM images were chosen because they were the only high-resolution CRISM images within the study region (Figure 1b), covering the Noachian Basement Group, that contained both L- and S-data (Murchie et al., 2007). In addition, five multi-spectral CRISM images (MSP and MSW) were used for the construction of a map west of Jezero and north of NE Syrtis due to lack of high-resolution CRISM images (see section 2.5). The 30 CRISM TRR3, 4 MSW, and single MSP images (Murchie et al., 2007) were processed using the CRISM Analysis Toolkit 7.4 in ENVI (Morgan et al., 2009). Data were converted to $\frac{I}{F^{*}\text{cos}(\theta )}$, as defined in section 2.2, using procedures described in Murchie et al. (2007). Subsequently, the data were atmospherically corrected using the volcano scan correction (McGuire et al., 2009) and projected (Morgan et al., 2009; Murchie et al., 2016). Minimal processing of CRISM images was performed, usually relying solely on pixel averages of regions of interests with spectra ratioed to a spectrally bland unit within the same column. In some cases, noise reduction was performed using methods by Pan et al. (2017). CRISM bandmaps were constructed using band parameters from Pelkey et al. (2007), Viviano-Beck et al. (2014), and Carter et al. (2013).

In addition, we calculated a custom band parameter for distinguishing LCP signatures, a spectral centroid, corresponding to the wavelength position of the maximum between 1 and 2 μm. The spectral centroid is defined as: $\text{Centroid}=\frac{\sum_{i=1}^{N}I_{i}\lambda_{i}}{\sum_{i=1}^{N}I_{i}}$. Here *i* refers to each of the *N* number of bands used for this calculation. *I* refers to the intensity of the reflectance value for each band, while *λ* refers to the wavelength value of each band. We used all bands between the fixed range of 1 to 2 μm for this calculation in order to track the position of maximum reflectance between the 1- and 2-μm absorptions. This band parameter was designed as we observed a minor change in LCP spectral characteristics correlating with geomorphology. The compositional significance of the LCP centroid was evaluated by investigating the centroid positions of previously published laboratory spectra of pyroxenes with different compositions (Klima et al., 2011). In addition, the centroid positions of calculated linear mixtures of LCP with regional dune materials, previously published laboratory Fe/Mg-smectite (Fox et al., 2019), and previously published Fe-rich glass spectra (Cannon et al., 2017) were compared with the Mars CRISM data.

When present, CRISM bandmaps and corresponding HiRISE were analyzed together in order to define subunits within the Noachian Basement. Detailed manual coregistration between CRISM and HiRISE was performed for 12 key locations (Figure 1). We evaluated the following characteristics in HiRISE in order to characterize Noachian Basement units/features: albedo, texture, HiRISE color, smoothness/roughness, relative crater densities, topographic expression, and thickness. Geological units were defined to be materials of the same lithology with a defined volume and clear contact with other units. Geomorphological features were defined to be materials of the same lithology or collection of lithologies with a singular geomorphological expression that did not have a clear contact with other units, for example, ridges and blocks, but rather appeared to be within units. Lastly, we use the term mineral deposits to categorize kaolinite-bearing bright materials (KBM; see section 3.2.7), as these could be identified via spectral characteristics but did not have consistent geomorphic or stratigraphic characteristics.

### 2.4. Stratigraphy and Structural Analyses

Stratigraphic analyses were based primarily on visual inspection of HIRISE digital elevation models (DEMs). We constructed one HiRISE DEM through SOCET SET (Kirk et al., 2009). This study also used a number of HiRISE DEMs covering NE Syrtis and the Mars 2020 Midway landing ellipse made available to the Mars 2020 Science Team by the Murray Lab at Caltech and processed through the National Aeronautics and Space Administration Ames Stereo Pipeline (Beyer et al., 2018). Furthermore, we constructed 6 HiRISE DEMs through the Ames Stereo Pipeline, primarily for visual inspection (Figure 1). All visual inspections were performed using OSGEARTH that renders HiRISE DEMs at full resolution. Measurements and elevation profiles were performed in ArcGIS. Subsequently, two of these HiRISE DEMs were used to calculate orientations (strike, dip, and angular errors) of layers within stratified parts of the Noachian Basement and the contact between megabreccia and this stratification. These orientation calculations were performed through the attitude software package developed by Quinn and Ehlmann (2019b).

### 2.5. Geological Map of Noachian Basement Accessible to a Mars 2020 Extended Mission

The geological units of the Noachian Basement Group were mapped with HiRISE data at 1:5,000 resolution for the area between NE Syrtis and the western Jezero rim that could constitute an extended mission area for Mars 2020 and that is within the safe Midway landing ellipse presented at the Mars 2020 workshops (Farley et al., 2018) (Figure 20). The map in the vicinity of the NE Syrtis ellipse was adapted from mapping of the Noachian Basement geomorphological units by Bramble et al. (2017) at 1:1,000 resolution. However, changes, additions, and reclassifications were made to the original Bramble et al. (2017) map in order to align the map with the specific geologic units defined within this study. Mapping of the megabreccia and megabreccia lithologies within this map was performed using methods of section 2.2. Because hyperspectral CRISM data are not available and the spatial resolution of CRISM multispectral data was too coarse for the scale of spatial variability of units in most cases, distinguishing between knobby parts of Mixed Lithology Plains Unit (MLPU) and LCP-bearing Plateaus Unit (LPU; see section 3.2) was not entirely possible and has been left ambiguous within the map. Low-resolution multispectral CRISM data (MSW and MSP) were used to look for strong LCP-signatures. Strong LCP-signatures were occasionally found and the area was subsequently mapped as LPU. However, the spatial resolution (~100–200 m/pixel) was generally too low to distinguish outcrops of the LPU that usually only have a lateral extent of a couple of hundred meters, so rover-scale investigations would likely reveal more geological unit diversity than that delineable from orbit. In comparison, the Blue Fractured Unit (BFU) was easily distinguishable due to its distinct texture and blue color.

## 3. Results

### 3.1. Megabreccia

Megabreccia occur within the Noachian Basement in outcrops of a single to thousands of <1- to 433-m-sized angular to subrounded blocks. These blocks have a variety of textures, but all have a sharp albedo and texture contrast with surrounding matrix materials. Outcrops with megabreccia blocks are usually highly eroded exposing a flat cross section of the original block. However, less eroded, protruding blocks do occur occasionally (Goudge et al., 2015).

#### 3.1.1. Megabreccia Map

**Figure 3.**
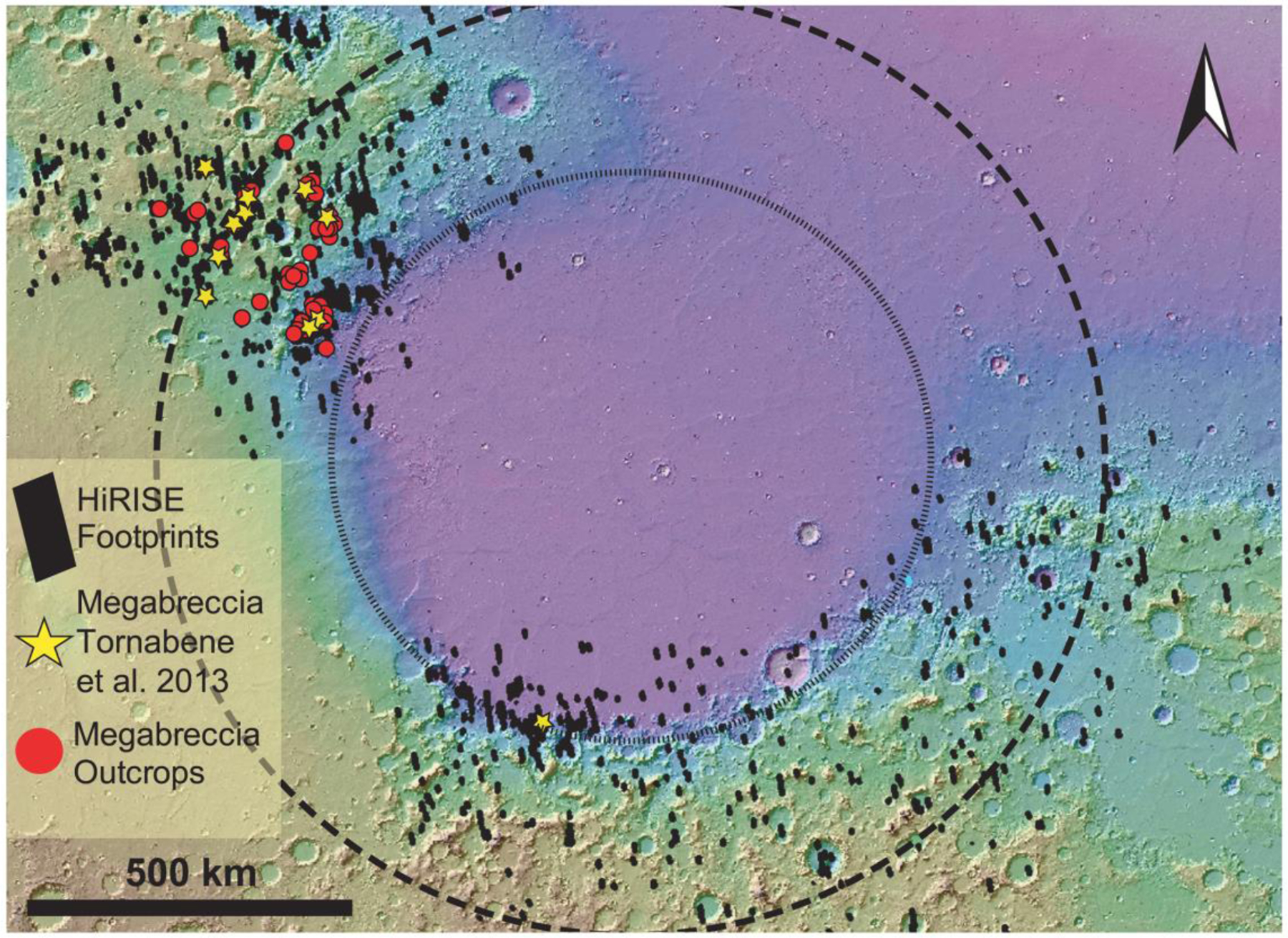
Map of all 173 megabreccia cataloged in this study within the Noachian Basement Group (red circles, overlapping at this spatial scale) along with megabreccia locations compiled in Tornabene et al. (2013) (yellow starts). Inferred inner and outer ring of Isidis basin from Figure 1 shown as black stippled lines. All analyzed HiRISE image footprints in black.

Our final map of megabreccia consisted of 173 megabreccia deposits within the NW part of the Isidis crater structure, assessed within 10^6^ km^2^ of HiRISE images (Figure 3). We only found megabreccia in the NW region between the putative outer and inner ring (Figures 1 and 3). Previous studies have observed spectroscopic signatures in the southern part of the Isidis impact structure, identical to the Noachian Basement and olivine-carbonate in the NW region of the study area (Bishop et al., 2013; Mustard et al., 2009). However, the exposure is considerably worse. We searched south and east of the Isidis basin structure, but due to thick, fine-grained covers within this region (see section 2.1), it remains indeterminate whether megabreccia are present. We did not positively locate any megabreccia in our search of ~80,000 km^2^ of HiRISE cover in the southern part of the Isidis structure (Figure 3). The prior detection by Tornabene et al. (2013) (Figure 3) is associated with Duvolo crater and, therefore, not necessarily associated with the Noachian Basement Group.

**Figure 4.**
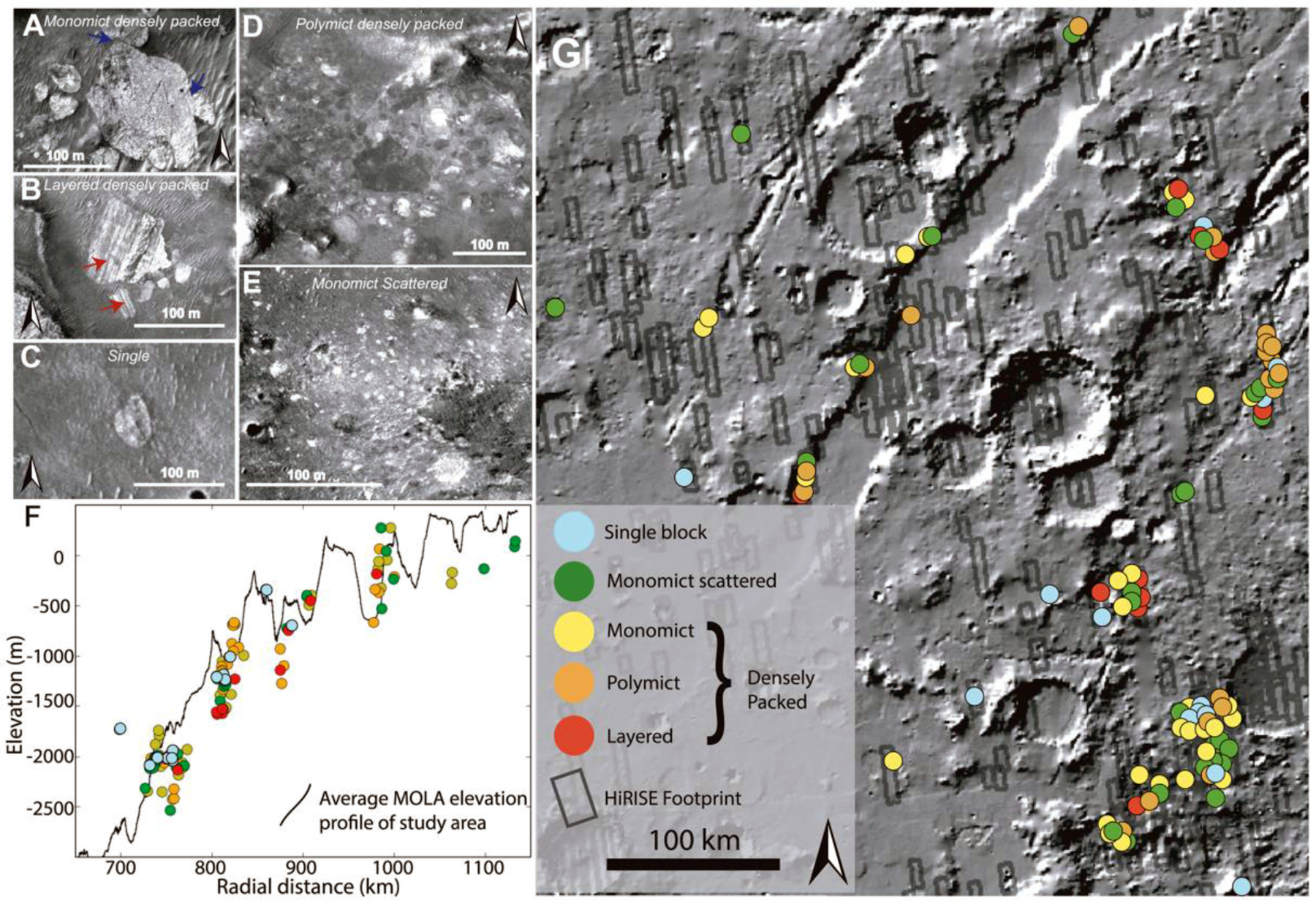
Examples of (a) monomict, densely packed megabreccias (ESP_033572_1995; Outcrop ID 64 in Data Set S1), (b) layered, densely packed megabreccia as indicated by red arrows (ESP_035062_1995; Outcrop ID 67 in Data Set S1), (c) single megabreccia (ESP_053523_1985; Outcrop ID 40 in Data Set S1), (d) polymict, densely packed megabreccia (ESP_037185_2010; Outcrop ID 50 in Data Set S1), and (e) scattered megabreccia (PSP_008861_2000; Outcrop ID 108 in Data Set S1). (f) Megabreccia outcrops of different textures plotted by radial distance from the center of Isidis basin and MOLA elevation. The black line in the background represents the average MOLA elevation profile of the study area. (g) Megabreccia outcrops of different textures plotted in plan view with MOLA background. All HiRISE footprints studied are outlined in dark gray.

We generated five classifications of megabreccia outcrops on the basis of block distribution and textural properties (Figure 4). “Densely packed blocks” are outcrops where blocks occur in contact with each other. “Scattered blocks” are outcrops where blocks do not occur in contact with each other, and “single blocks” are a single megabreccia block with no association to larger exposures (Figure 4). Second, densely packed blocks can appear “monomict” or “polymict,” depending on the number of distinct lithologies present based on albedo or HiRISE color properties (Figure 3). Third, certain polymict blocks exhibit layering with meters to tens of meter scale banding of material with alternating colors or albedos (Figure 4). The spatial distributions of these textures were investigated in 3-D and plan view as a function of radial distance to Isidis crater center and elevation (Figure 4). The different textural types of megabreccia had no obvious trends in their distribution and occur throughout the study area, particularly where eroded scarps provide a window in to the Noachian Basement (Figure 4).

**Figure 5.**
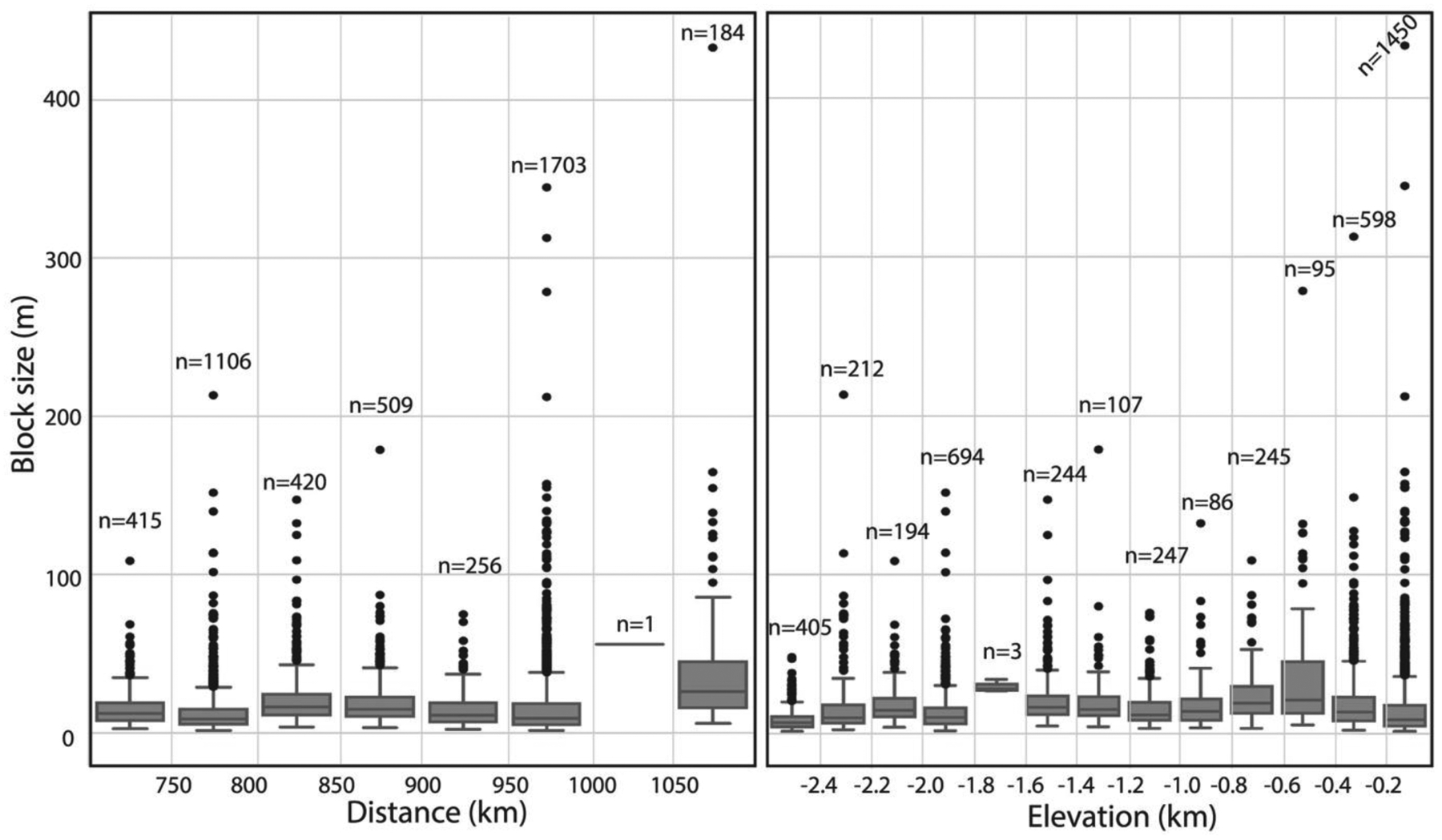
Boxplots of 4,600 megabreccia block sizes within each distance and elevation bin. The gray box encompasses the interquartile range (IQR) including the 25th (Q1) and 75th percentiles (Q3). The black line in the box indicates the median. Whiskers show lower (Q1–1.5 × IQR) and upper (Q3 + 1.5 × IQR) range of boxplots. Gray dots show all megabreccia points outside the lower and upper range. The number of megabreccia within each bin is denoted above each boxplot. Note that certain bins at 1,000–1,050 km and elevation of −1.8 to −1.6 km have too few megabreccia to construct proper boxplots and may be disregarded.

Megabreccia blocks have an overall size range of 1.3–433 m with a median of 11.5 m. Block sizes have similar characteristics (quartiles and ranges) at different distances from the crater center and elevation intervals with no apparent trends (Figure 5). We performed a Mann-Whitney U test in order to determine whether there were changes in the nonparametric distributions of block sizes within different bin intervals. Most pairs of block distributions achieved a *p* value <0.05 suggesting that block size distributions within bins are statistically different. Skewed normal and lognormal distributions were used to model distributions within each distance and elevation bins to investigate trends together with boxplots. Although the distribution parameters are statistically different, the median, quartiles, ranges, mean, mode, variance, skewness, kurtosis, and overall shapes of distributions of boxplots and distributions do not appear to exhibit any systematic changes with increasing distance to crater or elevation (Figure 5). In summary, each bin has a statistically different distribution compared to the others, but the differences were not systematic with increasing distance or elevation.

#### 3.1.2. Megabreccia Lithologies

**Figure 6.**
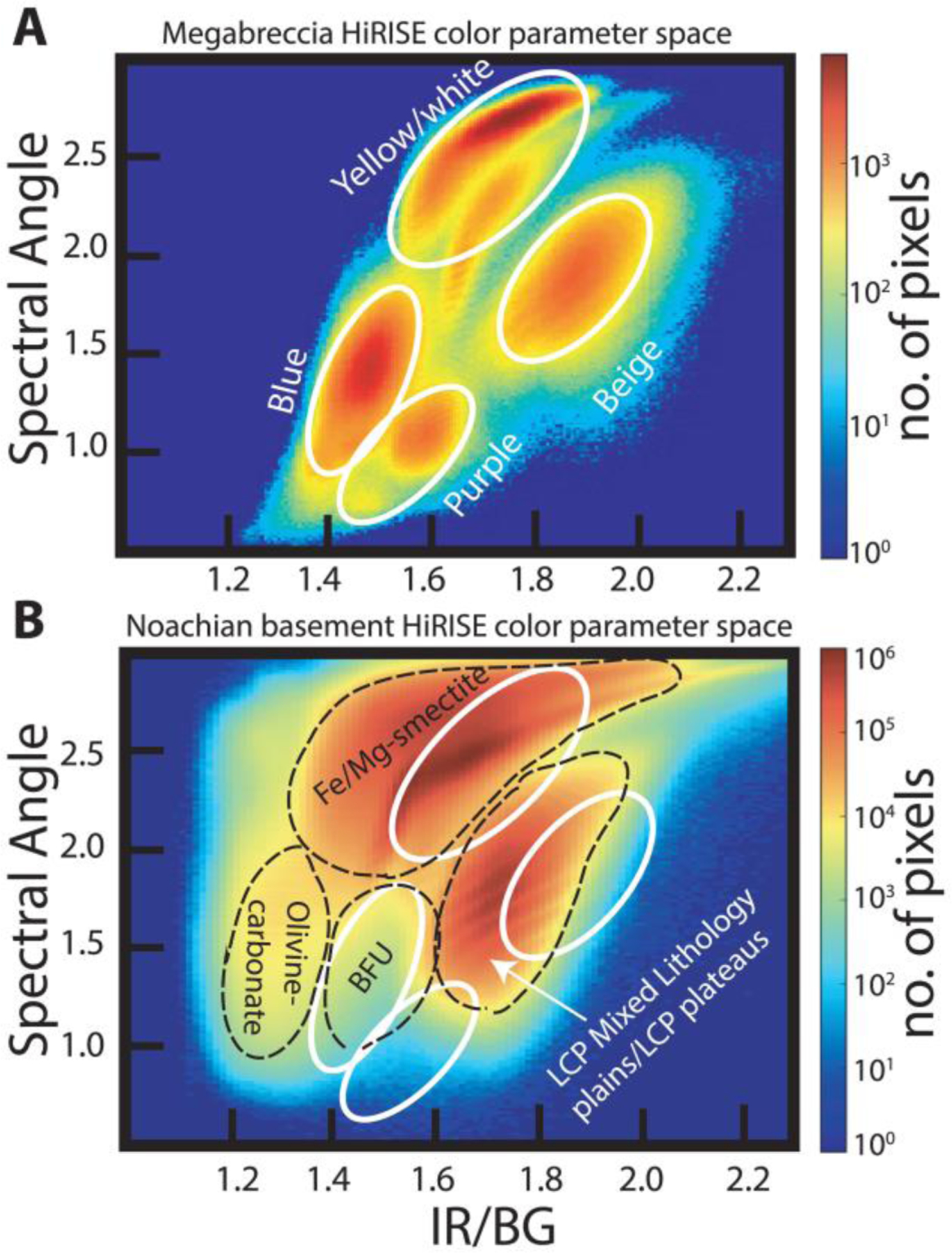
(a) The 2-D histogram of IR/BG band ratio and spectral angle from HiRISE color parameterization scheme from Figure 2. Data include only megabreccia blocks from eight different images that each contained a variety of different colored clasts. The megabreccia HiRISE color parameter space shows four main lithological clusters (white lines) that correlate with yellow/white, blue, beige, and purple visual colors from Figure 2. HiRISE images used are ESP_016153_2005, ESP_022601_1975, ESP_033572_1995, ESP_037185_2010, ESP_037541_2010, ESP_047049_2015, ESP_047339_1980, and PSP_002888_2025. (b) The 2-D histogram of IR/BG band ratio and spectral angle from entire HiRISE images containing Noachian Basement and olivine-carbonate units. Here, clusters (black stippled lines) were related to olivine-carbonate, Blue Fractured Unit (BFU), general Fe/Mg-smectite signatures in CRISM, and LCP-bearing Mixed Lithology Plains Unit and/or LCP-bearing Plateaus Unit. HiRISE images used are a portion of ESP_016153_2005, ESP_027691_2025, ESP_047049, and ESP_053655_1985.

Analysis of 12 separate exposures of megabreccia with CRISM reveal that LCP and Fe/Mg-smectite occur in megabreccia materials, as reported previously (Mustard et al., 2009) (Figure 2). Eight separate exposures of megabreccia from eight different HiRISE color images reveal that at least four different clusters of HiRISE color properties occur (Figure 6). We observed that one cluster always correlated with the visual color of blue, while another always correlated with the visual color of purple. The two last clusters correlated with yellow/white colors, although one of the clusters represented blocks of a more beige nuance (Figure 6a). The frequency distribution of color properties within the yellow/white blocks class may indicate two distinct subclasses (Figure 6a). Yellow/white megabreccia blocks were Fe/Mg-smectite bearing, while blue megabreccia blocks were LCP-bearing when CRISM data were available over large blocks, as also previously reported in Mustard et al. (2009) (Figure 2). Beige blocks did not have any CRISM coverage. In the few cases where purple blocks had CRISM coverage, the megabreccia did not occur in sizes large enough to obtain CRISM spectra (>18 m). Hence, beige and purple block lithologies are clearly distinct in HiRISE color but are unconstrained by VSWIR spectra.

Comparison between HiRISE color properties of Noachian Basement units and megabreccia (Figure 6b) shows that blue megabreccia blocks have similar HiRISE color properties and CRISM spectral characteristics as the Blue Fractured Unit (see section 3.2). Similarly, yellow/white megabreccia mostly share HiRISE color properties with Fe/Mg-smectite-bearing Stratified Basement Unit and Mixed Lithology Plains Unit on a regional scale (see section 3.2). The purple and beige megabreccia blocks are clearly distinct in color properties and are not represented in the larger basement units.

**Figure 7.**
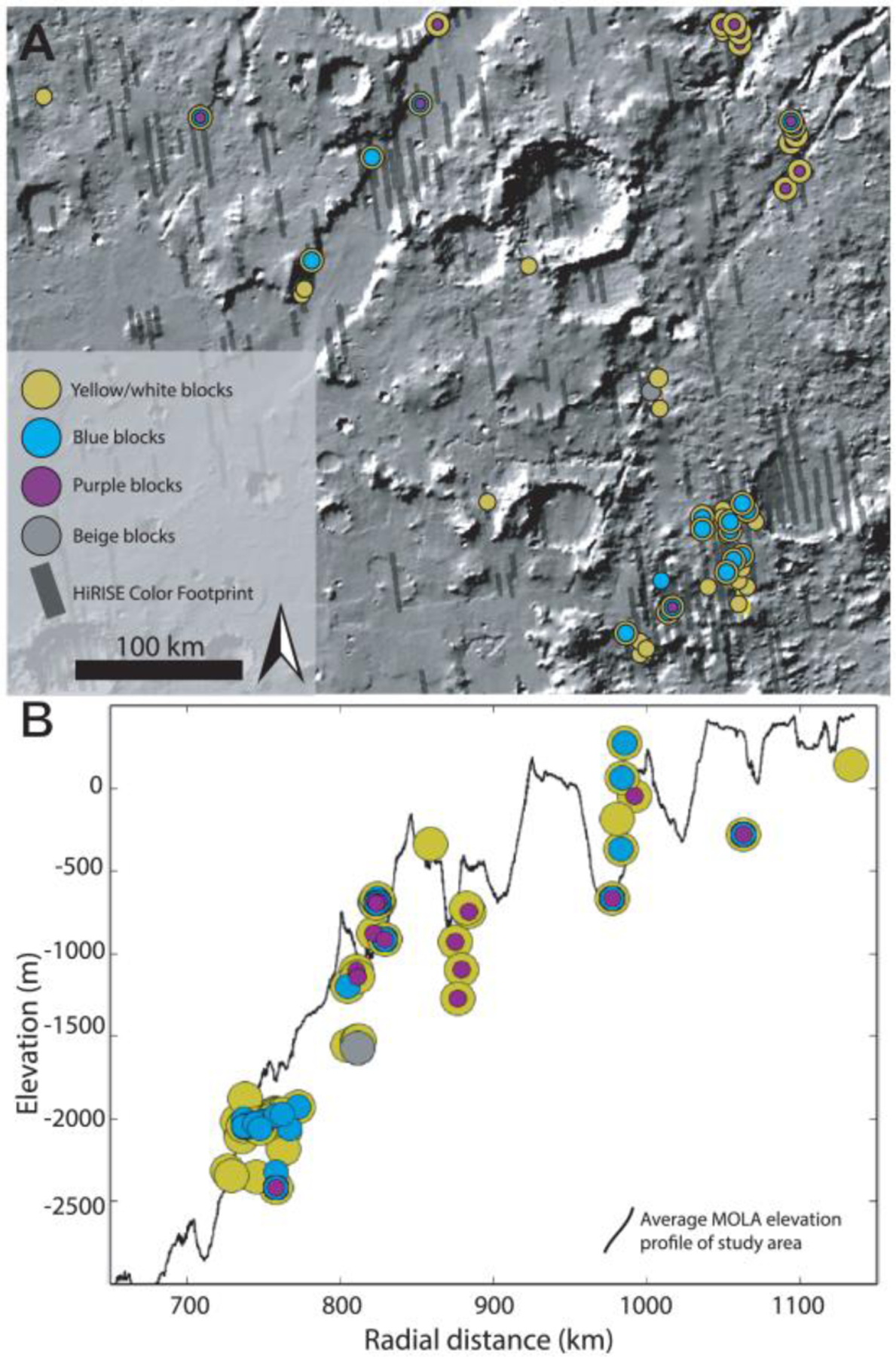
(a) Megabreccia outcrops of different visual HiRISE color (yellow/white, blue, purple, and beige colors) properties plotted in plan view with MOLA hillshade background. Outcrops with multiple colored circles represent outcrops that include blocks of multiple color properties. HiRISE color footprints in dark gray. (b) Megabreccia outcrops of different color properties plotted by radial distance and MOLA elevation. Outcrops with multiple colored circles represent outcrops that include blocks of multiple color properties. The black line in the background represents the average MOLA elevation profile of the study area.

The spatial distributions of the megabreccia color classes were investigated visually for spatial patterns or groupings (Figure 7). Most megabreccia exposures were dominated by yellow/white colored blocks. Beige megabreccia blocks were not easily distinguishable from yellow/white properties by visual inspection. In our quantification and classification maps, beige blocks only appeared in one out of the eight HiRISE color images processed within our study’s scope. Through quantified processing of all megabreccia-containing HiRISE color images by hand-mapping individual blocks, future studies could likely locate more beige megabreccia blocks. Generally, differently colored megabreccia appeared to occur directly juxtaposed next to each other throughout the study area where exposures of megabreccia are seen with no visual evidence of spatial groupings or changes with distance from the basin center or depth.

### 3.2. Geological Units of the Noachian Basement Group

**Figure 8.**
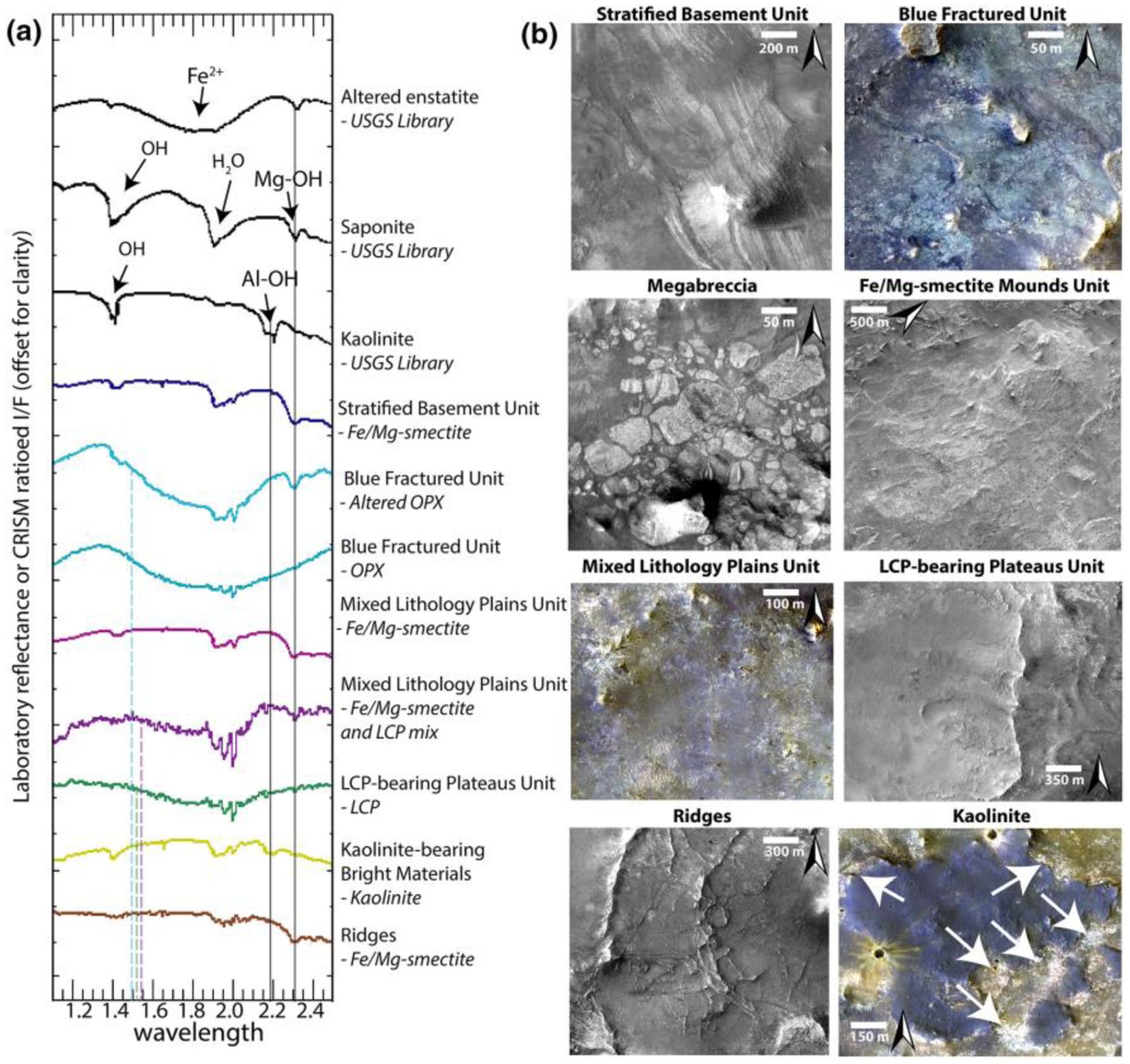
(a) CRISM spectra from regions of interest of geological units defined in this study. Black lines indicate the Fe/Mg-OH-related and the Al-OH-related vibrational absorption features. The blue, green, and purple dashed line indicates the position of the centroid for the Blue Fractured Unit (BFU), LCP-bearing Plateaus Unit (LPU), and Mixed Lithology Plains Unit (MLPU) respectively. Note the different character of the Fe^2+^-related absorption in LCP related to BFU, MLPU, and LPU as well as presence/lack of hydration features. Spectra from Stratified Basement Unit, altered BFU, BFU, LPU, Fe/Mg-smectite-bearing MLPU, and Fe/Mg-smectite- and LCP-bearing MLPU are from Projected Image FRT00009D44 and have center coordinates of 20°2′55.47″N 73°41′31.01″E, 20°2′51.05″N 73°41′2.78″E, 20°4′23.90″N 73°40′42.78″E, 20°0′33.98″N 73°38′8.69″E, 20°2′42.20″N 73°38′25.15″E, and 20°6′7.80″N 73°41′22.78″E respectively. Spectrum of kaolinite-bearing bright materials from Projected Image FRT0000CBE5 has center coordinates of 17°14′19.11″N, 76°21′18.01″E. Spectrum of ridges from Projected Image FRT0001997C has center coordinate of 17°33′36.95″N, 76°41′23.28″E. (b) Examples in HiRISE or HiRISE color of each of the eight geological units, geomorphic features, and mineral deposit of the Noachian Basement defined in this study (Table 3). Stratified Basement Unit from ESP_019476_2005, Blue Fractured Unit from ESP_053655_1985, megabreccia from ESP_039625_1995, Fe/Mg-smectite Mounds Unit from ESP_015942_1980, MLPU from ESP_015942_1980, LPU from ESP_016153_2005, ridges from ESP_027691_2025, and kaolinite from PSP_010206_1975.

Based on coupled HiRISE, HiRISE DEM, and CRISM analyses, we define five distinct geological units within the Noachian Basement Group: Stratified Basement Unit, Blue Fractured Unit, Fe/Mg-smectite-bearing Mounds Unit, Mixed Lithology Plains Unit, LCP-bearing Plateaus Unit; two geomorphological features, megabreccia (see section 3.1) and ridges; and one mineral deposit type, kaolinite-bearing bright materials. All geological units, geomorphological features, and mineral deposits of the Noachian Basement Group defined within this study are shown in Table 3, and characteristic spectra are shown in Figure 8.

#### 3.2.1. Stratified Basement Unit

**Figure 9.**
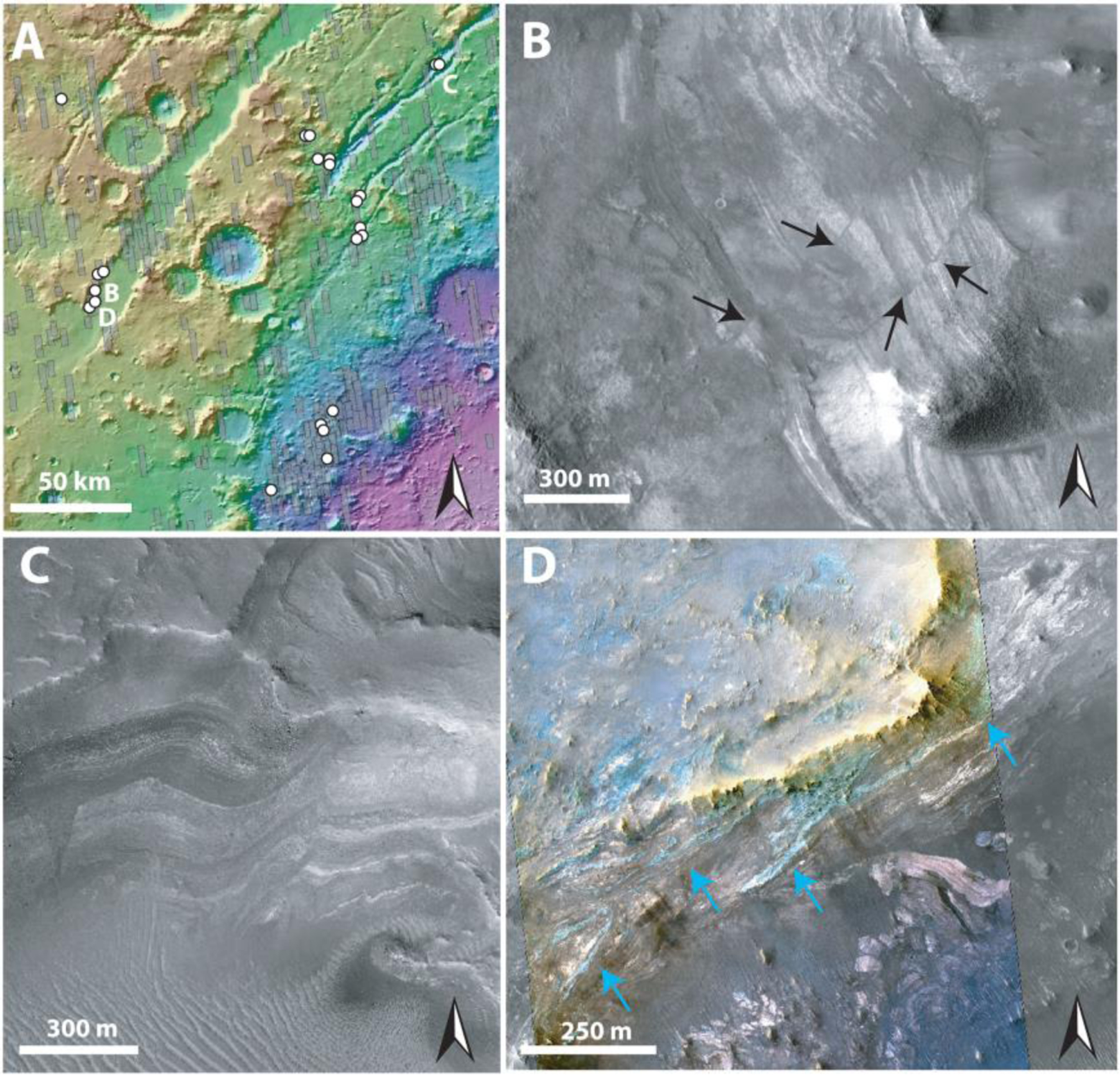
(a) Locations of Stratified Basement Unit (SBU) outcrops within the study area shown in white dots. Gray rectangles are HiRISE footprints. Locations B, C, and D refer to the position of panels (b)–(d). (b) SBU outcrop within the wall of the fossae. Block arrows show the location of two faults causing the offset of layers within the Stratified Basement Unit from HiRISE ESP_019476_2005. (c) Examples of similar SBU outcrops with multiple layers in northeast graben wall from HiRISE ESP_032227_2040. (d) Example of SBU within the wall of the fossae from HiRISE ESP_016153_2005. Blue arrows point toward bluish layers in HiRISE color. Here, Fe/Mg-smectite-bearing layers appear white. Note that bluish layers and SBU appear to be interlayered. Apparent folding in outcrops is the result of exposure and not a result of deformation (see section 3.2.1).

The Stratified Basement Unit (SBU) consists of materials of different albedos, layered at ~10-m scale. Its exposures extend over several kilometers, while individual layers can be traced over several hundreds of meters up to 1 km (Figure 9). The SBU is typically only exposed in graben walls, although a few examples occur in eroded flat terrains as well. Graben exposures of SBU typically underlie hundreds of meters of overlying units. A total of 19 different exposures of SBU was observed throughout the NW part of the Isidis impact structure (Figure 9) with horizontal extents ranging over 0.2–8 km. The total thicknesses of these layered packages range between 50 and 450 m. Single SBU exposures consist of between 6 and 20 layers with a range of layer thicknesses from 8–42 m. It is likely that certain layer boundaries are not resolved at HiRISE resolution. Nine of these 19 exposures of SBU had CRISM coverage and were found to be dominated by Fe/Mg-smectite compositions (Figure 8). The layers are often displaced along faults that formed after the deposition of the SBU in its current position (Figure 9b). In addition, the faults of the Nili Fossae graben itself crosscut the layers of SBU (Figures 9b–9d). As noted by Mustard et al. (2009), bluish units are occasionally within yellow/white layers of the SBU (Figure 9d). These units have the same spectral signature as the BFU (section 3.2.2) with minor Fe/Mg-smectite components in CRISM (Figure 8). Additionally, HiRISE color analyses show that bluish layered materials have color properties similar to the BFU cluster in Figure 6b, whereas all other SBU materials have color properties similar to the Fe/Mg-smectite cluster in Figure 6b.

Orientations of layers (Data Set S3) and contact segments were measured over exposures of several kilometers in Nili Fossae graben walls. The strikes of layers in the SBU (*n* = 60) were N-S strike range (300–58°) with a westward shallow dip with a range of 2–26° and a median 10°. A few anomalous exposures of megabreccia blocks underlie the SBU (Figure 15c). The contact segments (*n* = 6) between SBU and underlying megabreccia blocks had a similar N-S strike range (299–42°) with a westward shallow dip with a range of 7–14° and a median of 12°. In order to achieve an average of orientations for all layers and contact segments, we excluded high error fit data and stacked measurement segments for a single calculation (Quinn & Ehlmann, 2019b). The stacked solution for SBU orientation is a strike of 0° and westward dip of 4° (rake error, *θ*_*max*_=17°), while the contact between the SBU and anomalous underlying megabreccia is a strike of 19° and westward dip of 9° (*θ*_*max*_ = 8°). While some of the layers appear deformed or folded (e.g., Figures 9d and 15c), we determined using HiRISE ortho-photos draped over HiRISE DEMs, contour lines, and fitting of planes to layers that the apparent folding was a viewing geometry effect due to the curved nature of the exposure and overhead view.

#### 3.2.2. Blue Fractured Unit

**Figure 10.**
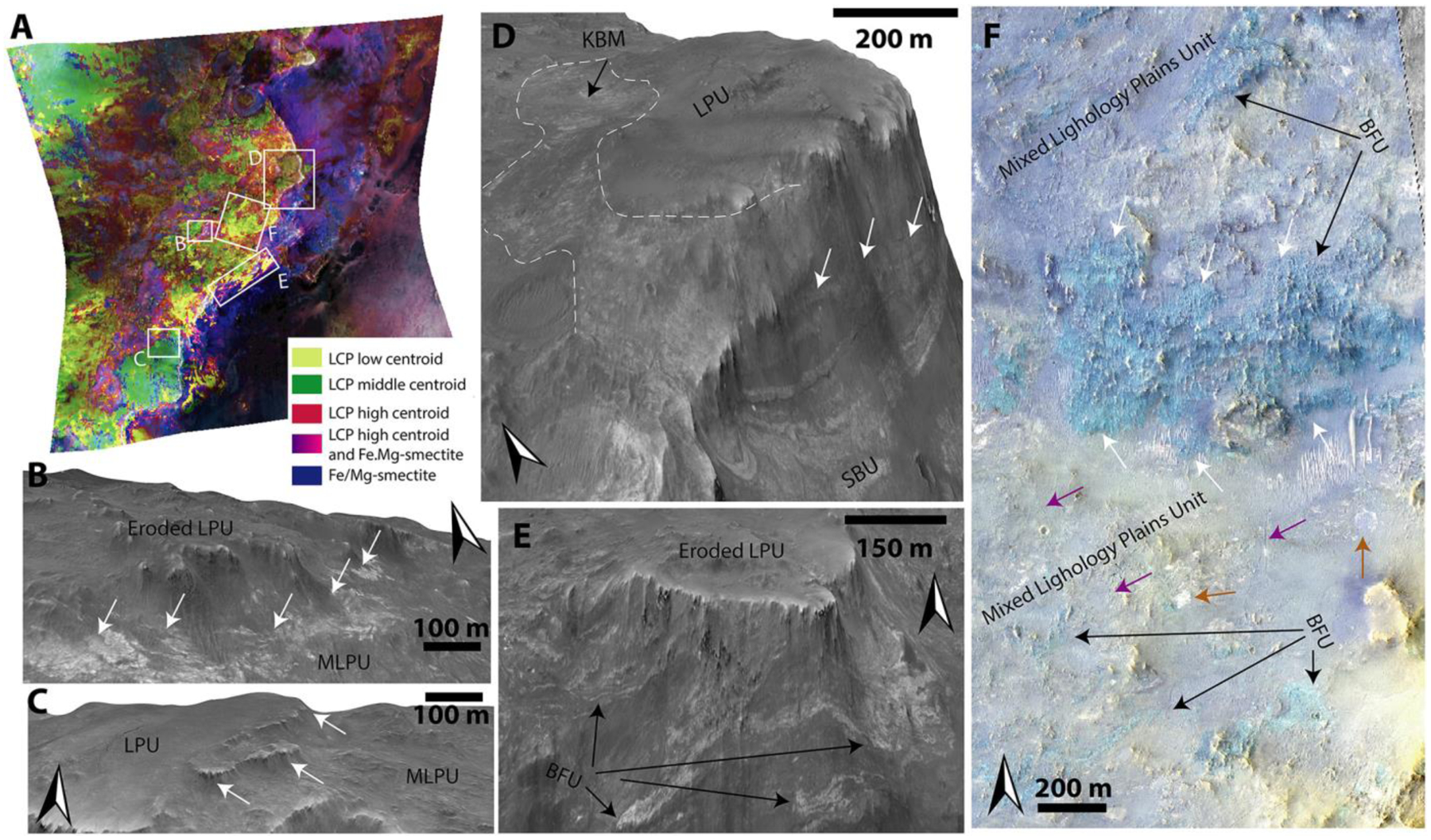
(a) Bandmap of CRISM image FRT00009D44 where R: LCPINDEX, G: LCP centroid custom parameter, and B: D2300. White rectangles show the locations of HiRISE images in panels (b)–(f). (b) Eroded remnants of LCP-bearing Plateaus Unit (LPU) that have a gradual contact between a particularly bright Fe/Mg-smectite-bearing part of Mixed Lithology Plains Unit (MLPU). (c) Example of LPU elevated compared to MLPU with well-defined edge of plateau (white arrows) with break in slope to MLPU (d) LPU elevated above Stratified Basement Unit exposed in the largest Nili Fossae trough. Additionally, a front of kaolinite-bearing bright materials occur on the edge of the LPU. (e) Eroded LPU overlying Stratified Basement with bluish layers. The same location in HiRISE color can be seen in Figure 9d. (f) Particularly resistant example of Blue Fractured Unit (BFU) forming a full outcrop that stands out compared to surrounding MLPU. Note that the mineralogical boundary between MLPU and BFU is sharp (white arrows). Several smaller angular blocks of BFU can be observed within the MLPU. Examples of putative megabreccia blocks eroded flat occur within the MLPU as well (brown arrows). Large fractures can be observed in the MLPU (purple arrows). All HiRISE images are from HiRISE ESP_016153_2005. All examples from HiRISE DEM have been vertically exaggerated by 3.

The Blue Fractured Unit (BFU) consists of a generally bright, highly fractured texture that appears primarily blue in HiRISE color; that is, it has diminished Near-infrared and Red albedo relative to typical Martian materials (Figures 8–10). The BFU is usually exposed as relatively small patches (~100–500 m) within the MLPU, but outcrops of several kilometers are also observed (section 3.2.4 and Figures 10f and 20). No craters are observed within these small units. The contact between the BFU and other parts of the Noachian Basement Group is sharp. However, due to the limited spatial extent and erosion of BFU, we do not see any of these contacts exposed in three dimensions, so the nature of the contact relationships is challenging to assess.

Compositionally, the BFU has a characteristic LCP-dominated spectrum with a very deep Fe^2+^-related absorption band (Figure 8). The spectral characteristics of the three LCP-bearing units defined within this study are distinguishable by the spectral centroid between 1 and 2 μm (Figure 8). Boxplots of the centroids show that the interquartile ranges for centroids of BFU, LPU, and MLPU are separated, although there is some minor overlap between the full ranges of centroids between the three different geological units (Figure 18). The median spectral centroid of this LCP band in BFU is 1.535 μm, which is the lowest, compared to other LCP-compositions in the Noachian Basement Group (Figure 8). Usually, the BFU does not appear to contain Fe/Mg-smectite in CRISM spectra. However, minor Fe/Mg-smectite signatures can occasionally occur (Figure 8). The distinct composition, textural expression, clear contacts, occasional volumetric and kilometer-scale outcrops, and a variety of different morphological expressions suggest that the BFU is a geological unit.

#### 3.2.3. Fe/Mg-smectite-bearing Mounds Unit

The Fe/Mg-smectite-bearing Mounds Unit (SmMU) occurs near the proposed landing ellipse in NE Syrtis and southwest of the proposed ellipse surrounded by the Syrtis lavas (Bramble et al., 2017; Ehlmann & Mustard, 2012; Quinn & Ehlmann, 2019a). Similar to geomorphic features also noted by Bramble et al. (2017), the SmMU always occurs as kilometer-scale diameter mounds protruding with a vertical elevation of up to around ~50 m above the surroundings, which are primarily composed of Fe/Mg-smectite from CRISM observations (Figure 12). These mounds usually have a sharp compositional, sometimes sharp topographical, and potentially stratigraphic contact (Figure 12e) with the generally flat-lying adjacent Mixed Lithology Plains Unit, suggesting that they form a geological unit with a singular geomorphological mode of occurrence (Figure 12).

#### 3.2.4. Mixed Lithology Plains Unit

**Figure 11.**
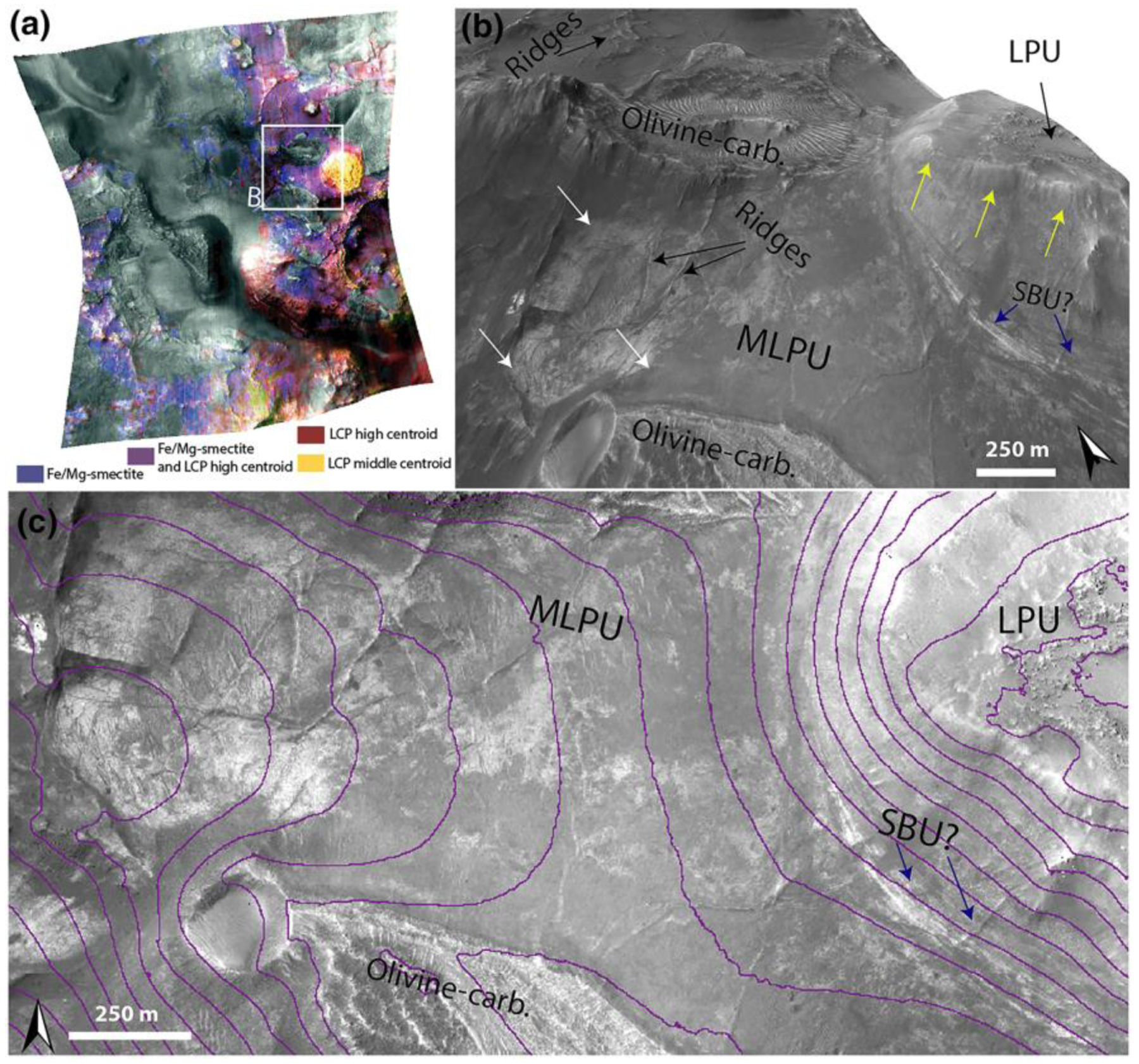
(a) Bandmap of CRISM image FRT0000B438 where R: LCPINDEX, G: LCP centroid custom parameter, and B: D2300. White rectangle shows the position of panel (b). (b) Example of contact between LCP-bearing Plateaus Unit (LPU) and Mixed Lithology Plains Unit (MLPU) (yellow arrows), where LPU is elevated compared to MLPU. Note albedo transitions in MLPU correlate roughly with stronger/weaker Fe/Mg-smectite signatures in the CRISM bandmap, although the textural change is diffuse and smooth (white arrows). Ridges appear to be crosscutting part of the MLPU (black arrows). We also observe a transition between MLPU to a putative outcrop of Stratified Basement Unit (SBU) underlying the LPU (blue arrows). HiRISE image from ESP_027691_2025. (c) The 2-D view of the same area as panel (b) with superimposed contour lines with 20-m intervals. This sections highlights the ambiguous contact between the Stratified Basement Unit and MLPU in smaller outcrops. Here, both units appear to occupy the same topographic interval.

**Figure 12.**
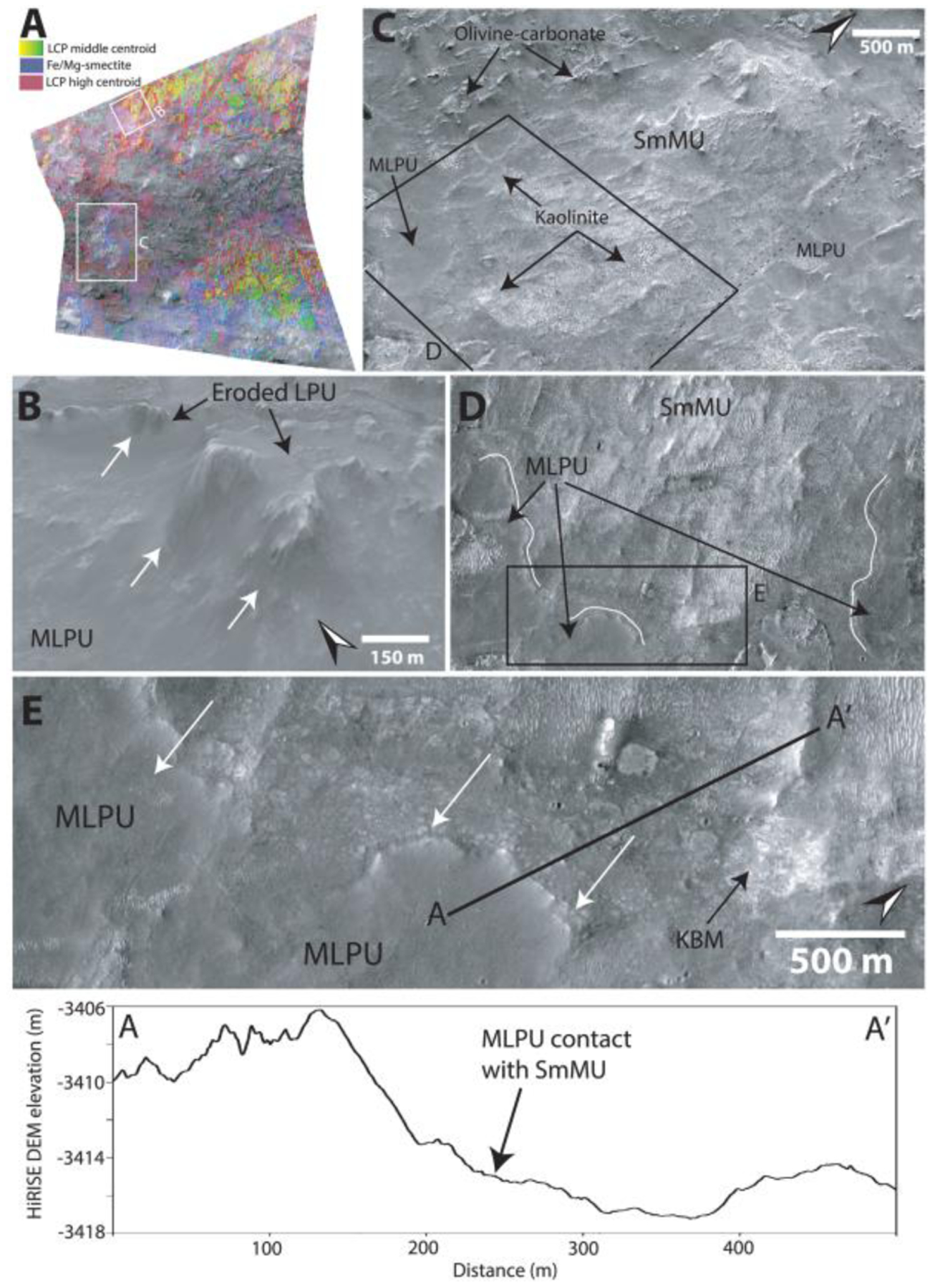
Bandmap of CRISM Image FRT0000161EF, where R: LCPINDEX, G: LCP centroid custom parameter, and B: D2300. White rectangles show the location of HiRISE images in subsequent panels. (b) Contact between eroded remnants of the LCP-bearing Plateaus Unit (LPU) and Mixed Lithology Plains Unit (MLPU) in NE Syrtis from HiRISE ESP_015942_1980. (c) Fe/Mg-smectite Mound Unit (SmMU) from HiRISE ESP_016931_1980. Stippled black lines are the image seam between HiRISE images ESP_016931_1980 and HiRISE ESP_015942_1980. Olivine-carbonate appears as minor mesas (large black arrows). Notice minor patches of kaolinite that occur at the edges of SmMU (large black arrows). Black rectangle shows position of panel (d). (d) Contact between SmMU and MLPU outlined in white lines. Black rectangle shows position of panel (e). (e) MLPU appears in sharp contact with the SmMU exhibiting a lobate morphology (white arrows). HiRISE DEM elevation profile line segment A-A′ shows the steep scarp of lobate MLPU contact to base of SmMU with 10-m elevation drop. All examples from the two HiRISE DEMs have been vertically exaggerated by 3.

The most extensive parts of the Noachian Basement Group consist largely of eroded plains (Figures 10–12). This Mixed Lithology Plains Unit (MLPU) is usually dominated by a spectral mixture of minor LCP and Fe/Mg-smectite components (Figure 8). The compositional transitions are diffuse in CRISM bandmaps, and there are no significant geomorphological distinctions between plains of different compositions at HiRISE-scale, although some albedo contrasts may occur (Figures 10–12). The LCP spectral signature has subdued band depths compared to the absorptions of BFU (Figure 8). The median spectral centroid of typical MLPU LCP is higher, ~1.549 μm, than other LCP-bearing units within the study area. Certain parts of the Mixed Lithology Plains Unit appear to be associated primarily with LCP, while other parts appear to be associated primarily with Fe/Mg-smectite. Mixing with Fe/Mg-smectite does not affect the position of the centroid much (Figure 18); see section 4.2. The full range of the LCP centroid is larger for MLPU compared to LPU-bearing Plateaus Unit and Blue Fractured Unit, which is unsurprising due to the vast size and heterogeneity of the unit (Figure 18). However, the interquartile ranges of the MLPU are clearly at longer wavelengths than the interquartile ranges of LPU-bearing Plateaus and Blue Fractured Unit (Figure 18). In addition, bandmaps utilizing the LCP centroid parameter easily delineate the morphological features that are characteristic for each unit (Figure 10a).

In general, the Mixed Lithology Plains Unit is characterized by laterally extensive plains with little topographic relief that occur in between megabreccia outcrops and other mound-, plateau-, and mesa-forming units. The MLPU is texturally smooth with the exception of occasional polygonal fracturing (tens of meters in length). In general, only smaller craters (tens of meters to hundreds of meters) or no craters at all are observed superposed on the MLPU. Large parts of the MLPU are featureless. However, the albedo and color of the MLPU can vary at the HiRISE-scale. Bright circular features and irregular bright patches are sometimes observed although these textural features appear to have similar elevations with no significant geological contacts. (Figures 10–12).

**Figure 13.**
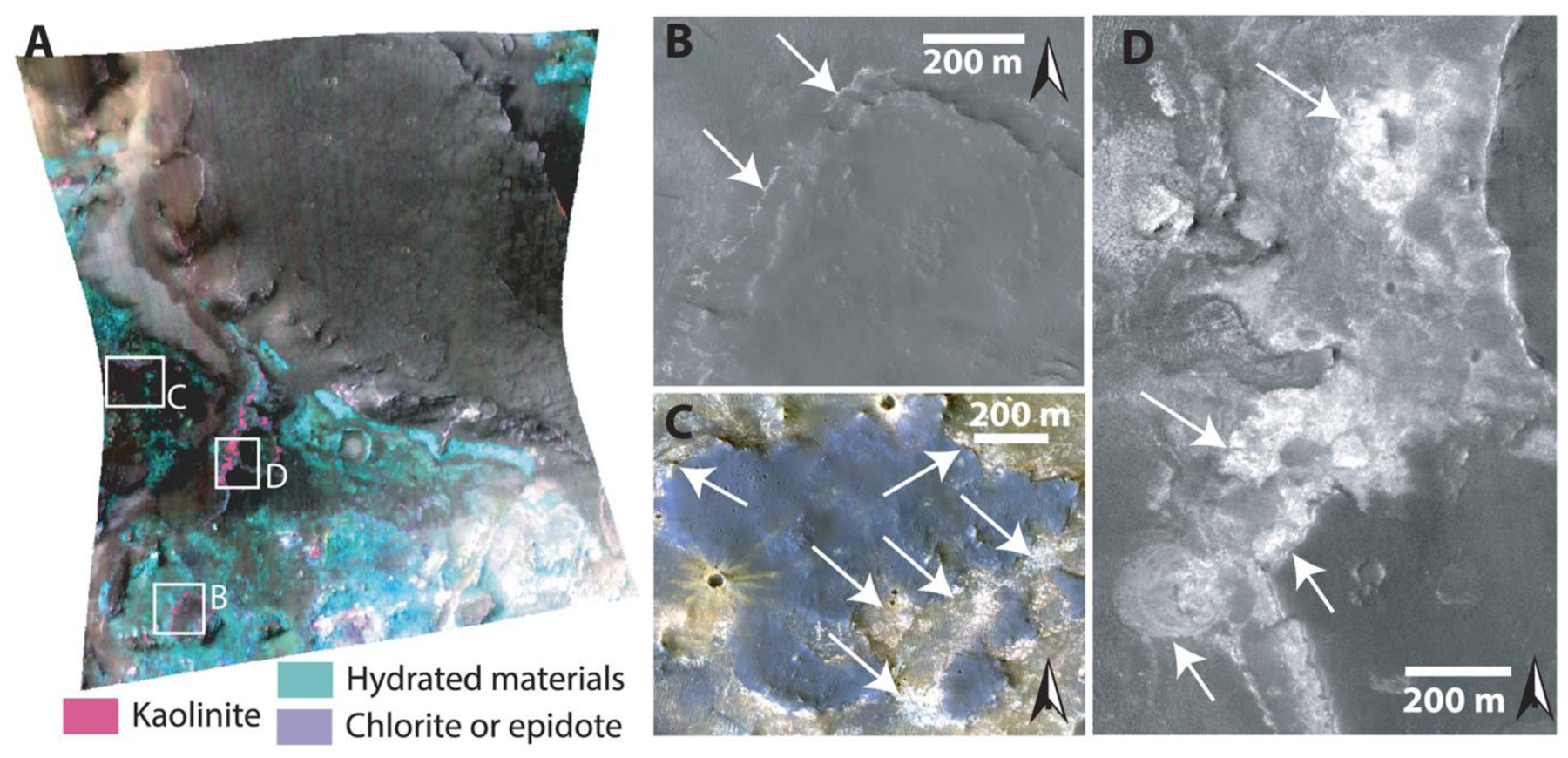
(a) CRISM Bandmap Image FRT0000CBE5 of parameters R:BD2.17 μm, G: D2.30 μm, and B: D2.32 μm from Carter et al. (2013). Pink color denotes kaolinite, turquoise color denotes materials containing hydrated minerals including both Noachian Basement and Olivine-carbonate Unit, and purple denotes the presence of either chlorite or epidote (Carter et al., 2013). (b) Kaolinite present as a layer overlain by a mafic mesa from HiRISE PSP_010206_1975. (c) Example of most general appearance of kaolinite as bright, irregular patches from HiRISE PSP_010206_1975. (d) Example of bright circular features of kaolinite from HiRISE ESP_022601_1975.

**Figure 14.**
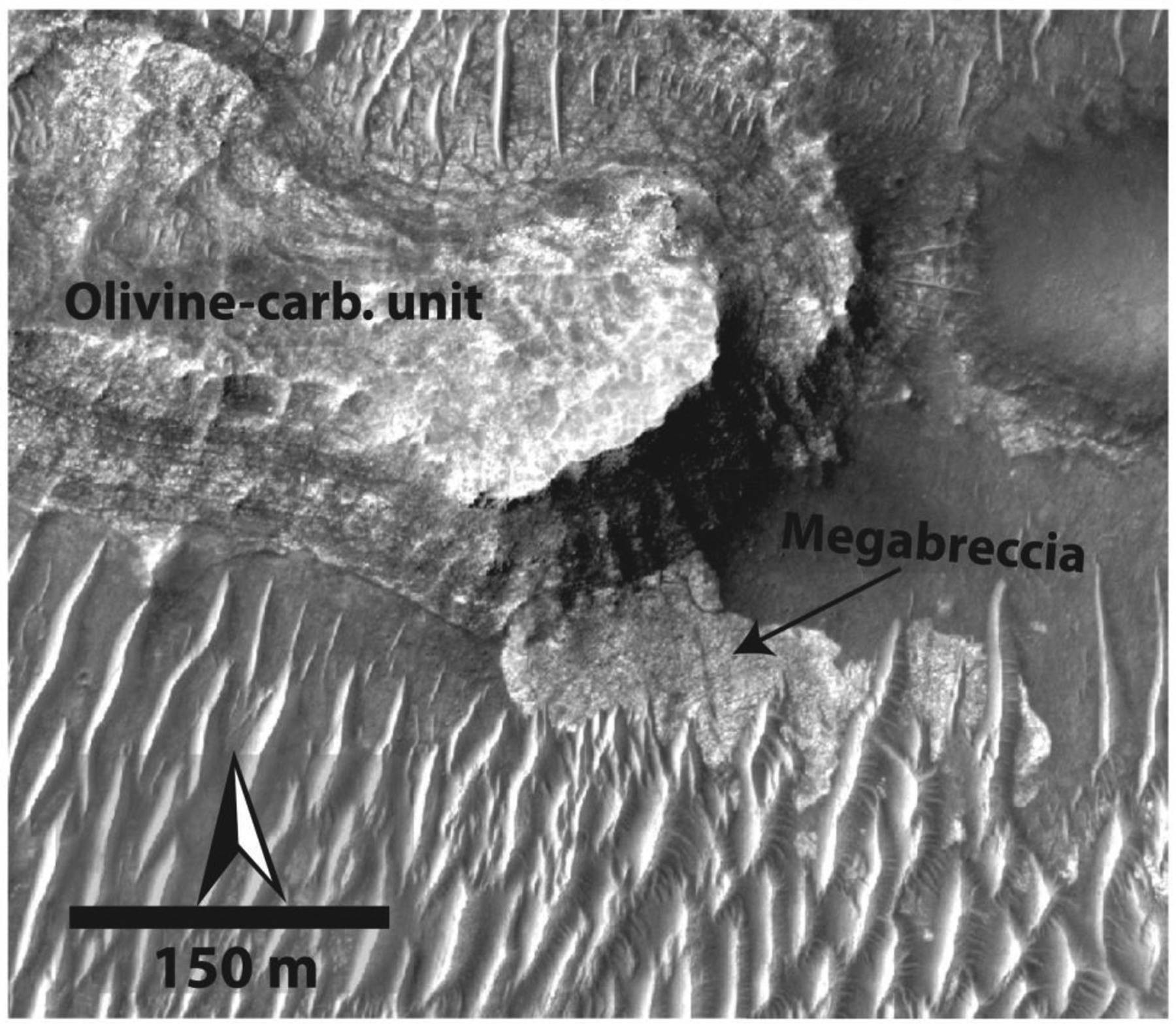
Megabreccia underlying olivine-carbonate unit from HiRISE ESP_035062_1995. There is no accompanying CRISM, but the Olivine-carbonate unit is defined in Goudge et al. (2015) and is consistent with its morphologic expression.

We determine that the Mixed Lithology Plains Unit should be defined as its own unit. This is based on the fact that MLPU has an identifiable lithology with spectral signatures (high centroid LCP and Fe/Mg-smectite mixtures) unique to this unit, a thickness, and contacts to the Stratified Basement Unit, LCP-bearing Plateaus Unit, and Fe/Mg-smectite-bearing Mounds Unit (see subsections of section 3.2; Figures 13 and 14).

**Figure 15.**
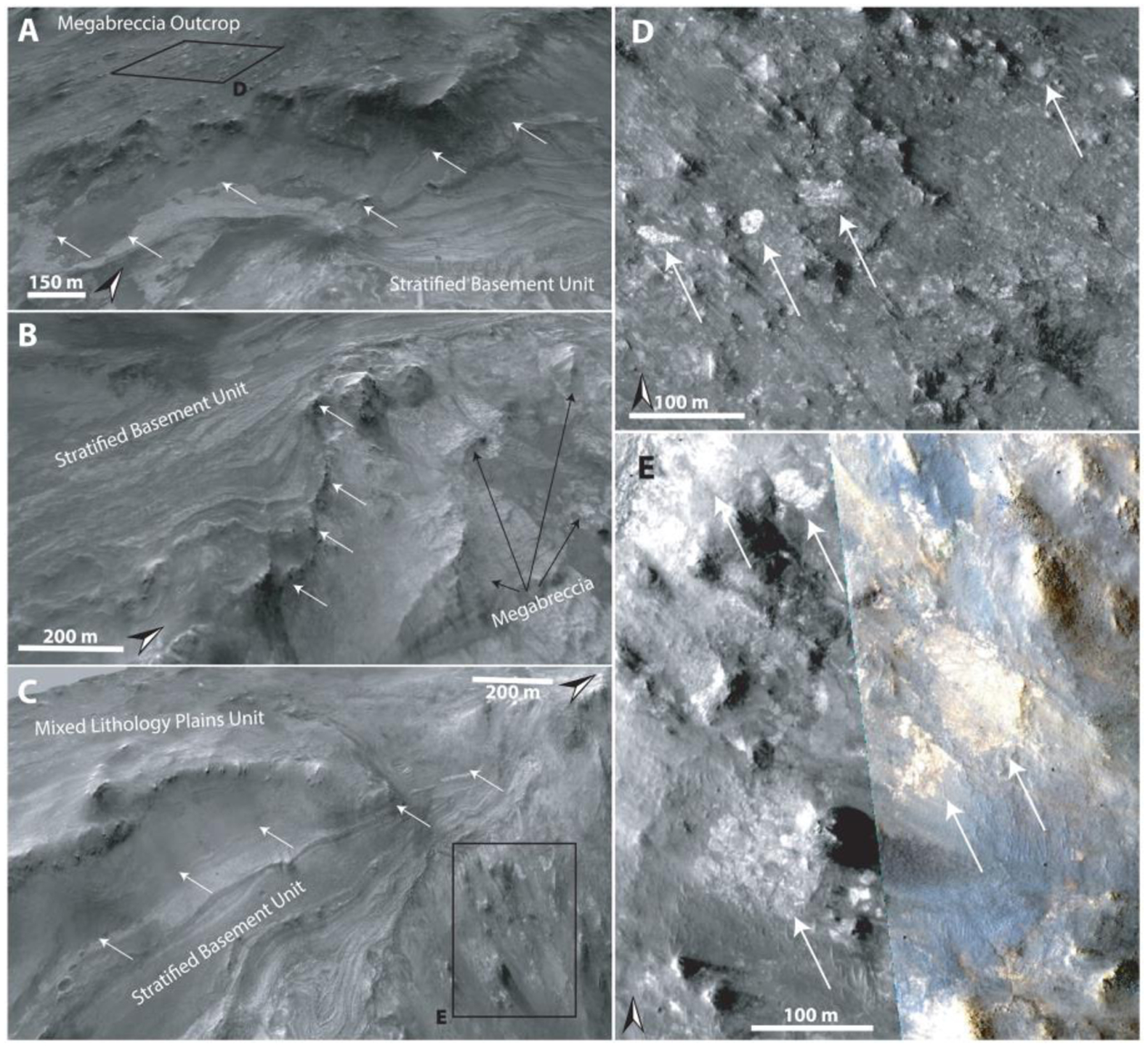
Noachian Basement exposures in the western Nili Fossae graben wall from HiRISE ESP_019476_2005. (a) Uppermost megabreccia outcrops within Mixed Lithology Plains Unit (MLPU) overlie Stratified Basement Unit (SBU), marked with white arrows. Black rectangle is showing location of panel (d). (b) Large megabreccia blocks appear to underlie SBU. The contact between SBU and underlying megabreccia is marked with white arrows. (c) MLPU overlie SBU. The contact between SBU and MLPU is marked with white arrows. Note smaller megabreccia blocks occur vertically below the SBU in the graben wall, although no clear contact is exposed. Black rectangle is showing location of panel (e). (d) View of megabreccia blocks (white arrows) present within the megabreccia exposure in panel (s). (e) View of megabreccia (white arrows) present within the megabreccia exposure in panel (c).

However, the occasional entrainment of blocks/patches of megabreccia and Blue Fractured Unit within the Mixed Lithology Plains Unit convolutes the distinction between these three units/features (e.g., Figure 10f). Megabreccia generally occur within the Mixed Lithology Plains Unit as blocks. Hence, megabreccia are considered to be a geomorphological feature contained within the MLPU. However, the Blue Fractured Unit is sometimes of large spatial extent with defined volume and contact relationships, necessitating its own unit definition (section 3.2.2). Completely unambiguous three-dimensional exposures of the Mixed Lithology Plains Unit are rare, but a few MLPU exposures in grabens have a thickness of tens of meters to hundreds meters with sharp transitions and contacts with underlying Stratified Basement Unit (Figures 10a and 15c). In a few exposures, the contact between MLPU and SBU is ambiguous but suggestive of MLPU surrounding the SBU (Figures 11b and 11c). Interpretation of the origin of the complex MLPU is further described in section 4.3.4.

#### 3.2.5. LCP-bearing Plateaus Unit

The LCP-bearing Plateaus Unit (LPU) generally occurs as elevated plateaus or mesas with a horizontal extent of hundreds of meters (Figures 10–12). The plateau surfaces are smooth and featureless with few craters. In some cases, the LCP-bearing Plateaus Unit is heavily eroded into smaller uneven, ridged surfaces even though they are still elevated from the Mixed Lithology Plains Unit. The LCP-bearing Plateaus Unit has a distinct LCP spectral signature from the two other LCP-bearing geological units. The LPU has a median spectral centroid of ~1.544 μm, intermediate between the Blue Fractured Unit spectral signature and the Mixed Lithology Plains Unit (Figure 8). In addition, the LPU does not exhibit any Fe/Mg-smectite or hydration signatures, not even occasionally (Figure 8). Typically, the LPU is 10–40 m thick, and there is a sharp break in slope at the contact with the underlying MLPU (Figure 10c). The slopes of LPU-associated plateaus are often obscured and usually have some debris cover but do not appear to shed boulders. In certain cases, the slopes may be highly eroded (Figure 12b). However, highly eroded parts of the LPU are not to be confused with the SmMU. Eroded parts of the LPU still maintain steep slopes and their characteristic LCP compositions, whereas the Fe/Mg-smectite-bearing mounds within SmMU are much larger (kilometer scale) with more gradual slopes and characteristic Fe/Mg-smectite compositions. Due to the fact that the LCP-bearing Plateaus Unit has a distinct composition, characteristic contacts to SBU and MLPU, and diminished susceptibility to erosion that results in well-formed plateaus, eroded plateaus, and ridged plateaus, we define the LPU to be a geological unit.

#### 3.2.6. Ridges

**Figure 16.**
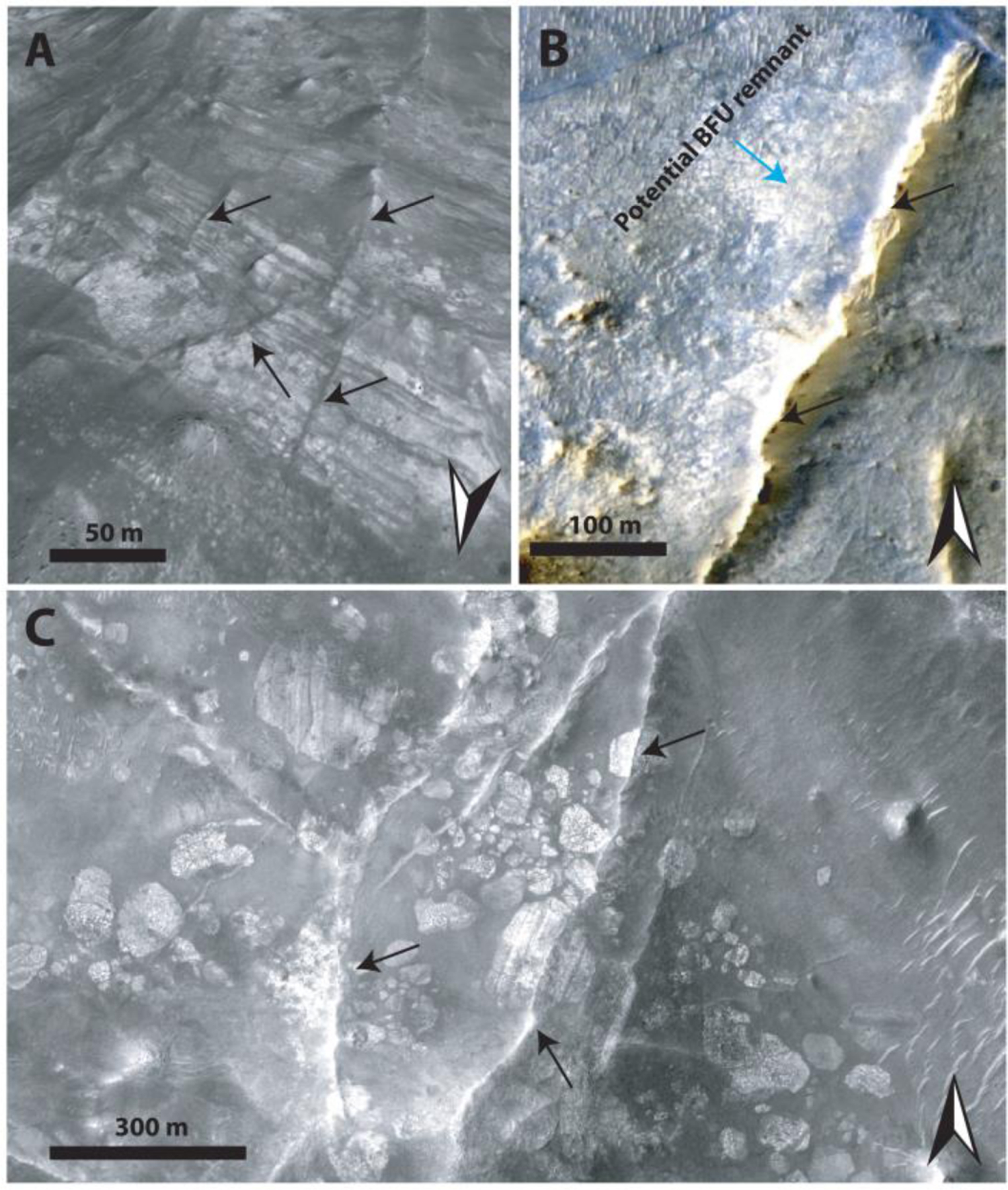
(a) Ridges (black arrows) crosscutting Stratified Basement Unit (PSP_002176_2025). (b) Ridge (black arrows) crosscutting a putative exposure of Blue Fractured Unit (BFU) (blue arrow) (ESP_052020_1985). (c) Ridges (black arrows) crosscutting megabreccia (ESP_033572_1995).

Geomorphological features that occur as ridges in the Noachian Basement Group have been characterized quite thoroughly geomorphologically and compositionally in previous literature (Saper & Mustard, 2013; Bramble et al., 2017; Pascuzzo et al., 2019). The ridges features refer to elevated curvilinear-linear features that crosscut most of the Noachian Basement Group units (Figures 11 and 16). Pascuzzo et al. (2019) noted that there are six different geomorphological types of ridges based on different geometric configurations. We refer to Pascuzzo et al. (2019) for images and descriptions of these. The ridges have all been observed to be composed of Fe/Mg-smectite-bearing materials that typically have weaker absorption minima compared to the host rock (Pascuzzo et al., 2019). We have observed ridges within or crosscutting SBU, BFU, megabreccia, and MLPU (Figure 16).

#### 3.2.7. Kaolinite-bearing bright materials

Kaolinite-bearing bright materials (KBM) have been described in various previous contributions that have noted they may be a weathering front or a unit of altered materials of distinct composition from the rest of the Noachian Basement Group (Bramble et al., 2017; Ehlmann et al., 2009; Ehlmann & Mustard, 2012; Mustard et al., 2009). In this study, kaolinite-bearing bright materials are observed to have a variety of geomorphological expressions. KBM are always bright in HiRISE and white in HiRISE color. They occur in small patches ~100 m across up to patchy exposures with kilometer-wide extent. These kaolinite-bearing bright materials sometimes have an irregular expression with diffuse or gradual contacts to surrounding materials (Figures 10d and 13c). However, our studies find that kaolinite-bearing bright materials can also occur as a ~5-m-thick layer in a mesa and as circular features with semiconcentric layering (Figure 13), although KBM are generally superficial (<2 m thick). The KBM classify neither as a geological unit nor as a geomorphological feature as they typically lack clear stratigraphic contacts, three-dimensionality of exposure, and a consistent identifiable geomorphological expression. Instead, we classify kaolinite-bearing bright materials as mineral deposits that are primarily identified based on composition in CRISM. The KBM appear to be at a higher stratigraphic level than Stratified Basement Unit (Figure 10d). We observe that KBM have formed within or on Fe/Mg-smectite Mounds Unit (Figures 12c), Mixed Lithology Plains Unit, and LCP-bearing Plateaus Unit (Figure 10d).

### 3.3. Stratigraphic Relationships

**Figure 17.**
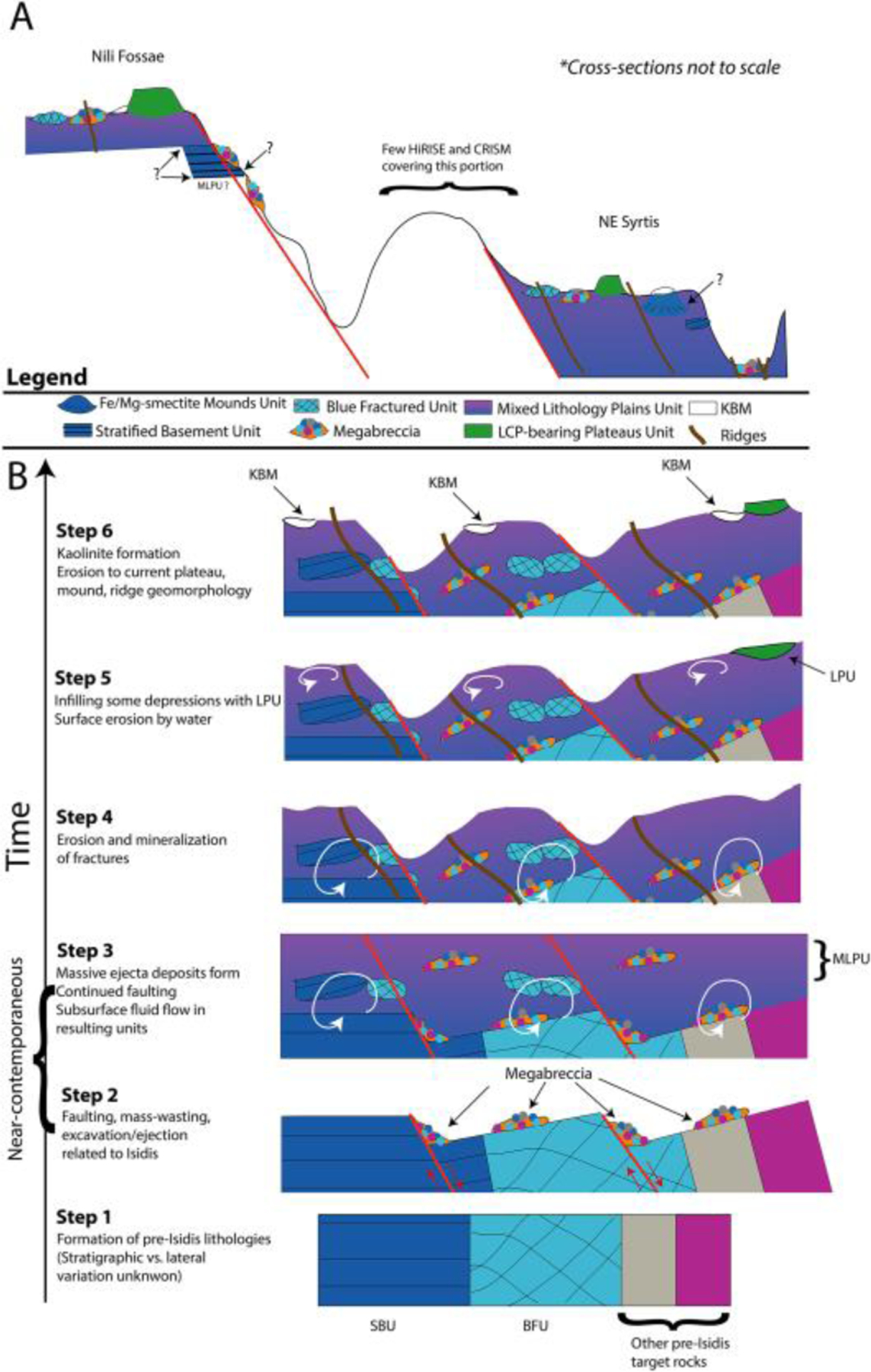
(A) Schematic regional stratigraphy of the Noachian Basement based on key locations in Nili Fossae andNE Syrtis. The lowermost units/features include Blue Fractured Unit (BFU),Stratified Basement Unit (SBU), megabreccia, Fe/Mg-Smectite Mounds Unit (SmMU) and Mixed Lithology Plains Unit (MLPU). The LCP-bearing Plateaus Unit (LPU) appear to be stratigraphically above these units. Ridges are observed to cross-cut all of the lowermost units. Kaolinite-bearing bright materials (KBM) has been observed to form on SmMU and MLPU. Question marks denote unresolved questions on the nature of individual contacts through lack of unambiguous contact exposures. These include the contact between MLPU and SBU, whether MLPU occurs underneath SBU in the Nili Fossae graben, megabreccia and SBU, SmMU and MLPU, and the diffuse transition between LCP-dominated and Fe/Mg-smectite-dominated parts of the MLPU. (B) Schematic of the preferred interpretation of the geological history of the Noachian Basement Group. Pre-Isidis lithologies include SBU, BFU, and other lithologies recorded within megabreccia blocks (e.g., beige and purple blocks). We neglect SmMU among these, as we do not understand its exact stratigraphic position. During Isidis basin formation mass-wasting and faulting leads to graben and megabreccia formation. Near-contemporaneous, excavation and ejection forms the MLPU that includes entrained deposits of pre-Isidis lithologies and megabreccia. Fluid flow within this system causes mineralization of fractures within these deposits. Erosion creates topography, and depressions are then infilled by the unknown LPU-forming process. Last, surface fluids are the cause for KBM-formation, and erosion leads to the modern plateaus/mounds/ridges expression of the LPU/SmMU/ridges.

The five geological units have defined contacts in HiRISE DEMs that appear to be systematic throughout the western part of the Isidis structure as examined at 14 key locations (Figure 1). A synthesis of our stratigraphic analysis has been visualized through a schematic cross section of the area (Figure 17). Stratified Basement Unit and Blue Fractured Unit always occur in lowermost parts of the basement stratigraphy. In a single out-crop, bluish materials similar to BFU in composition and texture are interlayered with SBU (Figure 9d). This would suggest that BFU and SBU are at a stratigraphically similar level and possibly related, although determining this would require additional HiRISE data along the Nili Fossae scarps.

Megabreccia also appear to be a geomorphological feature of similar relative emplacement age as the lowermost units. Megabreccia are always observed to clearly underlie the Olivine-Carbonate Unit, and we did not find any megabreccia outcrops where this contact is ambiguous (Figure 14). Additionally, no Olivine-Carbonate Unit compositional elements are found within megabreccia (Figure 6). However, while some megabreccia deposits overlie parts of the Stratified Basement Unit, other megabreccia appear to underlie parts of the Stratified Basement Unit (Figure 15). In certain cases, the contact between megabreccia and SBU is obscured as both materials have eroded to a flat plane. In these cases, it cannot be distinguished whether one is overlying the other or whether exposures are adjacent. Certain megabreccia blocks exhibit layering as described in Mustard et al. (2009) (Figure 4). As noted in Mustard et al. (2009), the scale of layering between layered megabreccia (meters scale) and SBU (tens of meter scale) does appear to differ. Hence, it is not clear if potential layered megabreccia precursor rock and SBU may be related to each other. For example, the Stratified Basement Unit could be larger sections of intact crust than the megabreccia, disrupted but preserved. In addition, blue megabreccia blocks appear to be similar to the Blue Fractured Unit (Figure 6). Hence, assuming the megabreccia blocks derive from BFU, the Blue Fractured Unit must have formed prior to formation of the megabreccia and is therefore stratigraphically below megabreccia deposits containing blue blocks.

The Stratified Basement Unit typically underlies the Mixed Lithology Plains Unit with a sharp contact in well-exposed kilometer-scale outcrops (Figure 15c). In a few cases, MLPU may appear to surround SBU (Figure 11), although these smaller outcrops cannot be interpreted with certainty. This could potentially suggest that minor parts of SBU are incorporated within MLPU similar to the entrainment of megabreccia and patches of BFU within the MLPU (Figure 10f). There is a clear stratigraphic relationship between LCP-bearing Plateaus Unit and Mixed Lithology Plains Unit. The LCP-bearing Plateaus Unit is always elevated and appears to overlie MLPU with a diffuse and often covered contact (Figures 10–12). Additionally, no megabreccia are observed within or in contact with the LCP-bearing Plateaus Unit as megabreccia generally occur at a lower topographic and stratigraphic level.

The youngest features in the geological sequence of events are kaolinite-bearing bright materials and ridges. Ridges directly crosscut Stratified Basement Unit, Blue Fractured Unit, megabreccia, and Mixed Lithology Plains Unit (Figures 11 and 16). However, no contact has been observed between ridges and LCP-bearing Plateaus Unit nor ridges and kaolinite-bearing bright materials, so their relative stratigraphic relationships are still uncertain. Kaolinite-bearing bright materials typically appear topographically higher than both Mixed Lithology Plains Unit and Stratified Basement Unit, although no stratigraphic contact is clearly observed (Figure 10d). In certain cases, KBM occur in the same plane as Mixed Lithology Plains Unit with a diffuse contact. We also observed that KBM have formed with a similar irregular expression and diffuse contact on eroded parts of the LCP-bearing Plateaus Unit and Fe/Mg-smectite-bearing Mounds Unit (Figures 10d and 12), suggesting that they have a younger stratigraphic age. Megabreccia also appear to be unaffiliated with kaolinite deposits (Figure 2) except for one block in the Jezero crater rim (see section 4.6), although kaolinite cannot be readily distinguished from Fe/Mg-smectite in the HiRISE color classification scheme.

The most stratigraphically inscrutable geological unit is the Fe/Mg-smectite-bearing Mounds Unit. It does appear older than kaolinite-bearing bright materials (Figure 12). However, the outcrops of this unit primarily occur in NE Syrtis and have no observed or resolvable contact with the Stratified Basement Unit, Blue Fractured Unit, megabreccia, and LCP-bearing Plateaus Unit. The contact between Fe/Mg-smectite-bearing Mounds Unit and Mixed Lithology Plains Unit is quite sharp compositionally (Figure 12). In several locations, we observe an elevation drop of ~10–20 m from the top of the MLPU to the bottom of the SmMU (e.g., Figure 12e). Furthermore, the terminal parts of the MLPU appear lobate at these contacts suggesting that the MLPU may be embaying the SmMU (Figure 12). This would make SmMU stratigraphically older than MLPU.

## 4. Discussion

### 4.1. Defining the Noachian Basement Group: Comparison to Previous Studies

In previous studies, the Noachian Basement has generally been treated as a single mineralogically and geomorphologically heterogeneous unit. Here, we define a basement group with five geological units, two geomorphological features, and a mineral deposit based on compositional, textural, and stratigraphic contacts.

Mustard et al. (2009) noted that certain parts of the Noachian Basement were LCP-, Fe/Mg-smectite, or kaolinite-bearing, which is similar to the spectral diversity that we have observed (Figure 8). The LCP originally described in Mustard et al. (2009) is similar to the Blue Fractured Unit and LCP-bearing Plateaus Unit materials, which we clearly delineate as two different units formed at different times and occupying different stratigraphic positions. Fe/Mg-smectite from Mustard et al. (2009) includes both the Stratified Basement Unit and Mixed Lithology Plains Unit. At 5-m/pixel Context Camera-scale, Goudge et al. (2015) divided the Noachian Basement in Dusty Massive Basement, Altered Basement, and Ridged Altered Basement, which we all consider Mixed Lithology Plains Unit, with and without crosscutting ridges. By contrast, our unit definitions make finer compositional distinctions and ridges are considered features. The geomorphological units defined in Bramble et al. (2017) are the most similar to our geological units. Bramble et al. (2017) defined Smooth and Knobby Plains Units similar to the Mixed Lithology Plains Unit in this study and Raised Linear Ridges Unit similar to Mixed Lithology Plains Unit with crosscutting ridges. In addition, Bramble et al. (2017) defined Crustal Mounds/Large Crustal Mounds Units that encompass the two distinct LCP-bearing Plateaus Unit and Fe/Mg-smectite-bearing Mounds Unit defined in this study. Our study subdivides these because their mineralogy, physical expression, and stratigraphic position are distinct. It was suggested that kaolinite-bearing parts of the Noachian Basement formed later than Fe/Mg-smectite-bearing parts (Carter et al., 2014; Ehlmann et al., 2011, 2009). Likewise, it has been suggested that the ridges are a younger feature forming after the Isidis impact (Pascuzzo et al., 2019). Both are confirmed within this study. However, a series of new enigmatic geomorphological expressions including circular layered structures suggest that further study is needed to explain the full diversity in geomorphological expression of the kaolinite-bearing bright materials and that these may not be a discrete geologic unit, hence our classification here as a mineral deposit (Figure 13).

### 4.2. Origin of Spectral Differences Between LCP-Bearing Units

**Figure 18.**
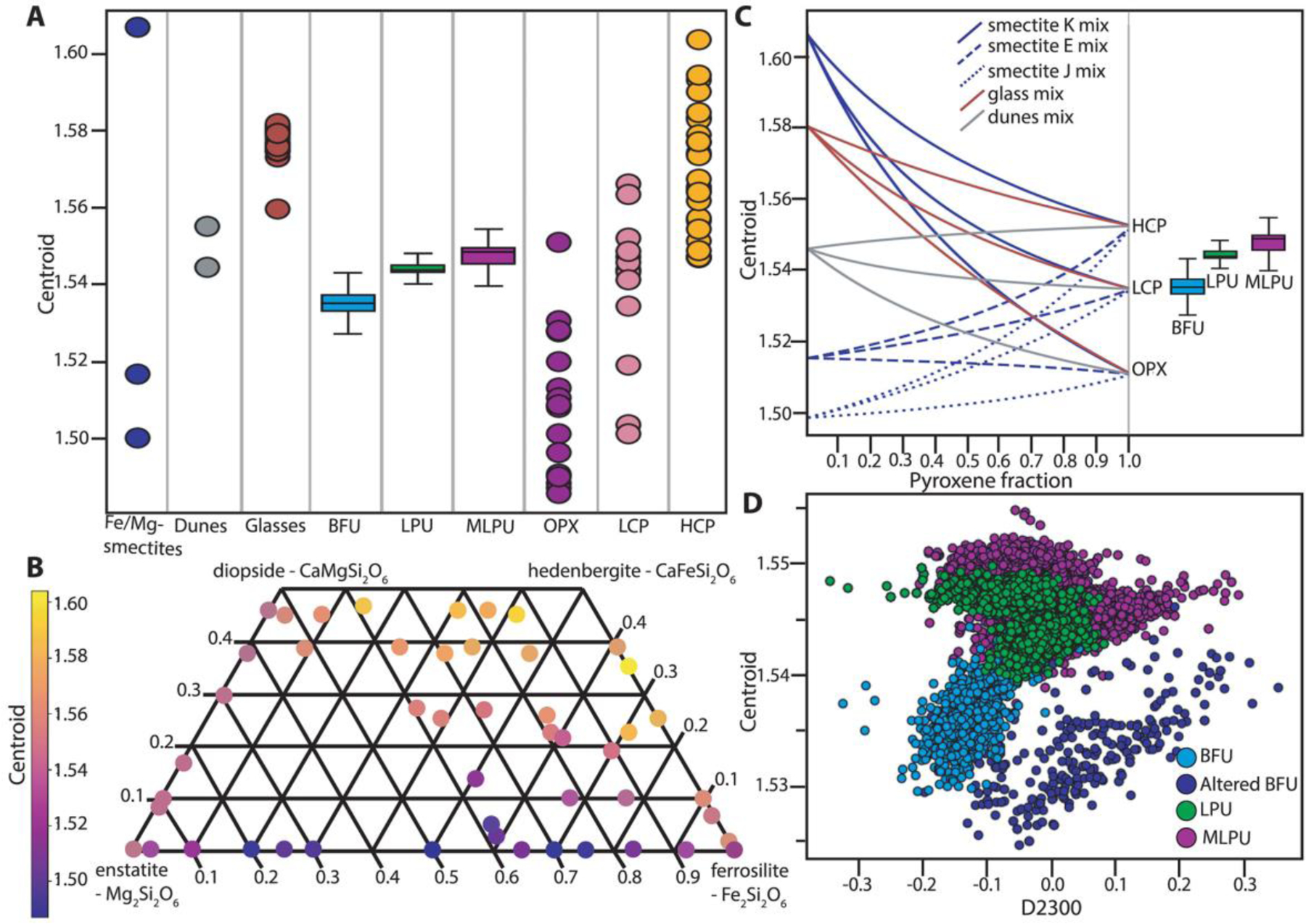
(a) Centroid positions between 1 and 2 μm for a variety of materials. Dots show Fe/Mg-smectites of three different compositions from Fox et al. (2019) (navy), dunes from the study area (gray), Fe-bearing glasses from Cannon et al. (2017) (maroon), orthopyroxenes (OPX) from Klima et al. (2007) (magenta), low Ca-pyroxenes (LCP; red), and high Ca-pyroxenes (HCP; burnt orange) from Klima et al. (2011). Box plots show centroid of Blue Fractured Unit (BFU), LCP-bearing Plateaus Unit (LPU), and Mixed Lithology Plains Unit (MLPU) from two different CRISM scenes in NE Syrtis (FRT000161EF, Figure 12) and Nili Fossae (FRT00009D44, Figure 10). (b) Centroid positions of pyroxenes with different compositions from Klima et al. (2007, 2011) plotted in pyroxene quadrilateral. Note that an increase in Ca content generally causes a higher centroid position. (c) Mixing lines for pyroxenes of three different compositions (OPX, LCP, and HCP) with the three different Fe/Mg-smectites from Fox et al. (2019), a representative glass from Cannon et al. (2017), and representative dune composition. Smectite K, E, and J refers to smectite of compositions Ca_0.23_[Fe_2.51_Al_0.26_Mg_0.12_][Si_3.51_Al_0.49_]O_10_(OH)_2_, Ca_0.40_[Fe^III^_1.06_Mg_0.93_Al_0.15_][Si_3.70_Al_0.30_]O_10_(OH)_2_, and Ca_0.37_ [Fe^III^_0.27_Mg_2.31_Al_0.08_][Si_3.60_Al_0.40_]O_10_(OH)_2_ respectively. Boxplots of BFU, LPU, and MLPU are shown to the side for easy comparison. (d) Plots of centroid position and the D2300 band parameter from Pelkey et al. (2007) for pixels of BFU, altered BFU, LPU, and MLPU from the two different CRISM scenes.

The centroid between 1 and 2 μm roughly indicates pyroxene compositions as the centers of the 1- and 2-μm Fe-related absorption bands shift when the compositions of pyroxenes change (Figures 18a and 18b). Orthopyroxenes (OPX) and LCP have lower wavelength centroids than high-Ca pyroxenes (HCP). However, other materials with Fe-related absorptions such as Fe-bearing smectites and Fe-bearing glasses may affect the centroid, if mixed with pyroxenes. In addition, dunes in the study area have Fe-related absorptions, and different bedrock-sand proportions within pixels could give rise to a shift in the centroid. From investigation of calculated linear mixtures with Fe/Mg-smectites (Fox et al., 2019), glasses (Cannon et al., 2017), sand from the study area, and three different pyroxene compositions (OPX, LCP, and HCP; Klima et al., 2011), it appears that mixing with Fe-bearing glasses is likely to affect the centroid position (Figure 18c). Higher glass content gives rise to higher centroid position. In contrast, mixing with sand does not give rise to much change in the centroid position (Figure 18c).

Mixing with Fe/Mg-smectites can give rise to increase or decrease in centroid position depending on smectite composition (Figure 18c). We observe slight correlation between the centroid position and the D2300 band parameter that evaluates the depth of a Fe/Mg-smectite-related absorption at 2.3 μm (Pelkey et al., 2007) within altered BFU (Figure 18d). This suggests that some higher centroid positions within BFU could be driven by mixture with Fe-rich smectites. However, large parts of BFU, MLPU, and all of LPU do not appear to have correlated centroid position and D2300 parameter value. In addition, we observed no correlation between centroid position and BD1900 parameter value. This suggests that mixing the Fe/Mg-smectite is not the primary control on LCP centroid position changes between the three LCP-bearing units.

Hence, the major changes between the centroid position of Blue Fractured Unit, LCP-bearing Plateaus Unit, and Mixed Lithology Plains Unit are most likely related to changes in pyroxene compositions or glass content. In this case, BFU would have the least Ca-containing pyroxenes or the least Fe-bearing glass content. The MLPU would have the highest Ca-containing pyroxenes or the highest Fe-bearing glass content. A single ultramafic or basaltic Martian meteorite can contain a variety of pyroxenes, including both pigeonites and augites, and different levels of glass contents (Papike et al., 2009). Therefore, it is unclear from a petrogenetic perspective what causes the difference in LCP between the 3 units from an orbital scale. However, the distinct stratigraphic contacts and morphological variability in combination with differing pyroxene composition and/or changing proportions of glass content points to a different origin or change in depositional/emplacement regime of the three LCP-bearing units.

### 4.3. Isidis Impact Processes

#### 4.3.1. Megabreccia Formation Mechanisms

Megabreccia are formed through many processes such as impact cratering, volcanic caldera collapse, tectonic processes, mass wasting processes, and glacial activity. The size of megabreccia blocks, reaching ~400 m, their distribution within a region of Isidis-related concentric ring grabens, and the presence of compositional and textural elements similar to the surrounding Noachian Basement favor formation by the Isidis impact (Figures 1 and 3). Proposed ice-related processes in Isidis Planitia during 3–2.8 Ga (Guidat et al., 2015; Souček et al., 2015) and glacial features in Nilosyrtis (Johnsson et al., 2019) have no association with megabreccia.

**Figure 19.**
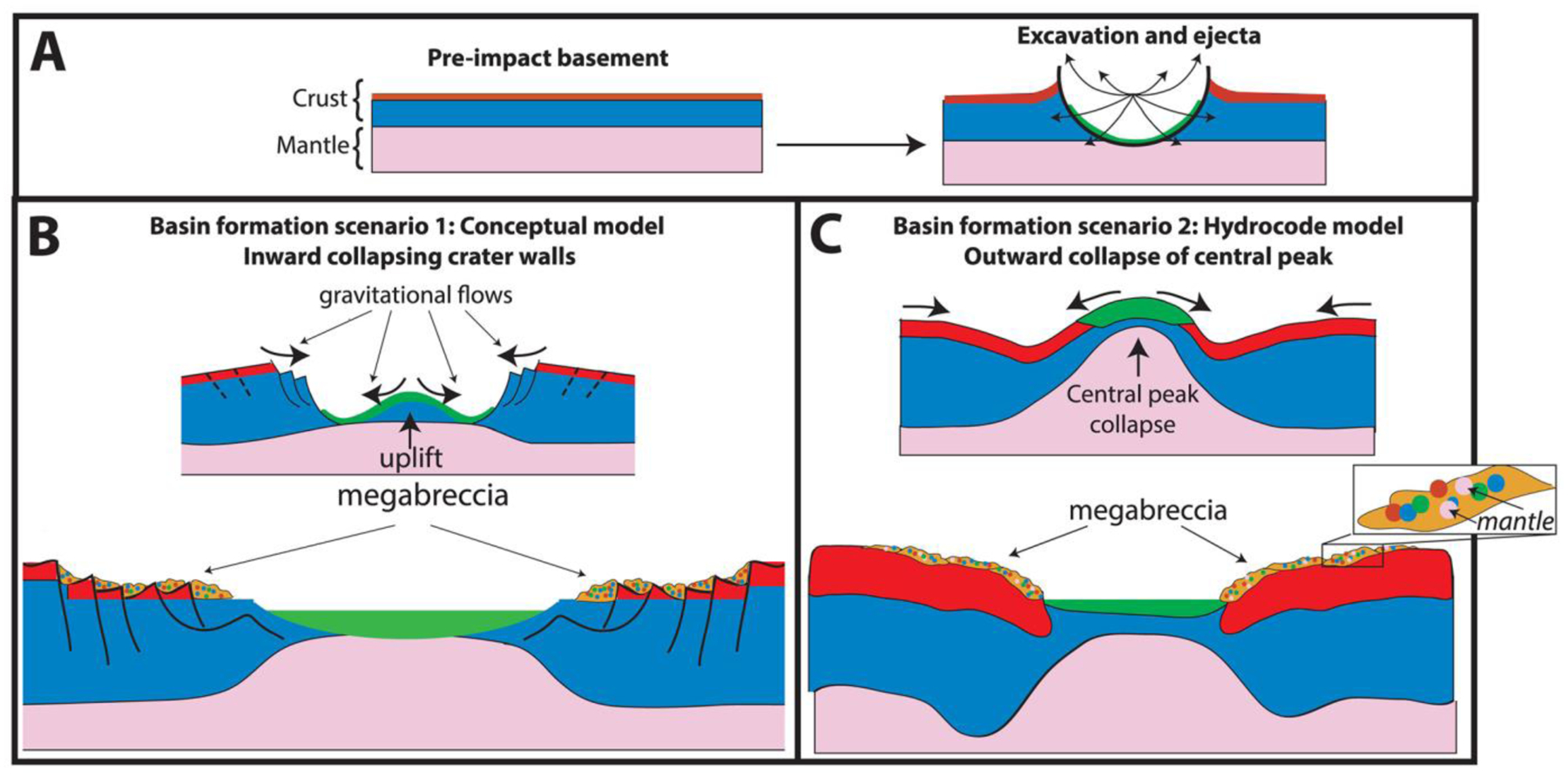
(a) Schematic showing a two-layer crust (red and blue layers) and mantle (pink layers) that is impacted, creating a transient crater and ejecta during the excavation stage. (b) One of several possible basin-forming scenarios where collapse of the transient is inward (Baker et al., 2016). This is presumably related to a central uplift and inward collapsing walls through faulting and graben formation. Black arrows signify the predominant directions of which gravitational flows may occur. Following the inward collapse, the impact structure would consist of a series of faults and grabens associated with primarily shallow crustal megabreccia from gravitational flows. Impact melt sheets (green) are also expected to have formed, predominantly within the central basin area. (c) Another of several possible basin-forming scenarios where collapse of the transient crater occurs outward (Baker et al., 2013). From hydrocode model results, we may expect the central peak to collapse outward. The putative flow associated with this collapse is possibly able to form megabreccia, which would be more deeply derived.

Impact cratering is observed to produce impact megablocks or megabreccia (meter to 100-m-scale-sized breccia blocks) in both simple and complex craters on Earth (e.g., Hörz, 1982; Osinski et al., 2005; Vishnevsky & Montanari, 2007), the Moon (e.g., Mustard et al., 2011; Stöffler et al., 2012), and elsewhere on Mars (e.g., Caudill et al., 2012; Grant et al., 2008; Tornabene et al., 2013). In particular, megabreccia are associated with ballistic ejecta, melt sheet and melt flows, crater floor and peak fracturing, and gravitational flows in association with crater collapse, fall back, and modification (Table 1). Our observations are most compatible with formation through gravitational flows associated with transient crater collapse due to the observed extent of megabreccia, the high heterogeneity of megabreccia materials, the large block size, and lack of distance dependency for megabreccia block sizes. In addition, we do observe some rounding of megabreccia blocks in between primarily angular blocks. Data from terrestrial landslides and avalanches suggest that occasional subrounding/rounding may occur through abrasion processes (Dufresne et al., 2016; Krieger, 1977). Mass wasting deposits in certain cases exhibit inverse grading due to kinetic sieving (Gray & Hutter, 1997; Gray & Thornton, 2005), which we did not observe (Figure 5). Megabreccia cannot be exclusively related to tectonic processes associated with faulting and graben formation because >100 megabreccia outcrops are unrelated to any graben/fault structures of the Nili Fossae (Figure 4), but faulting and slumping can be potential mechanisms for creating gravitational flows during transient collapse as discussed in section 4.3.3 (Figure 19).

Other formation processes can be excluded based on the extent of megabreccia, as megabreccia are observed outside the proposed inner ring but within the proposed outer ring of the Isidis impact basin (Figures 1 and 3). Crater floor/peak fracturing and a primary melt sheet (see section 4.2.3) would form within the inner ring, while ballistic ejecta and ejecta-associated melt flows would have an extent outside the outer ring (Barlow, 2005; Osinski, 2006; Osinski et al., 2011; Weiss & Head, 2014). In the case of a melt flows and melt sheet origin, one might expect megabreccia to be associated with melt flow structures, such as a melt matrix, melt injections, pseudotachylitic textures, and/or lobate flow structures. Additionally, one might expect block sizes to be dependent on distance from the crater center if formed through ballistic ejecta (Oberbeck, 1975), and we did not observe such a relationship (Figure 5).

#### 4.3.2. Megabreccia Lithologies and Relationship to the Basement

Quantitative investigation of HiRISE color properties showed at least four different lithologies within the megabreccia. From parallel analysis of CRISM spectra, the blue megabreccia with LCP materials are similar to the BFU within the Noachian Basement Group (Figure 6), and yellow/white materials are similar to other Fe/Mg-smectite-bearing materials in the Noachian Basement Group (Figure 6). This indicates that blue megabreccia blocks were potentially sourced from Isidis target rock similar to BFU. Yellow/white megabreccia lithologies could potentially have been sourced from any of the older Fe/Mg-smectite-bearing units (SBU or Fe/Mg-smectite-bearing Mounds), but the exact relationship to Fe/Mg-smectite-bearing Noachian Basement Group units are undetermined, as all materials with Fe/Mg-smectite signatures plot similarly in the examined parameter spaces on a regional scale (Figure 6). In contrast, beige and purple megabreccia materials appear to be different from any surface-exposed regional Noachian Basement units, suggesting that these megabreccia blocks are pre-Isidis lithologies not represented in the Noachian Basement Group and are Pre-Noachian or Early Noachian materials.

#### 4.3.3. Testing Impact Models: How Do Transient Craters Collapse?

Traditionally, two different models for the formation of peak-ring basins and their transient crater collapse have been considered (Baker et al., 2016; Morgan et al., 2016). The main difference between these two different models is the nature of the central uplift and its collapse (Baker et al., 2016; Morgan et al., 2016). Conceptual models based on observational evidence suggest that the transient cavity may inwardly collapse along a series of faults (Baker et al., 2016), giving rise to massive mass wasting from the outside inward (Figure 19). Additionally, we may expect uplift in association with the central peak of the basin that could also cause mass wasting. In contrast, hydrocode models of Orientale (Johnson et al., 2016) and Chixulub (Collins et al., 2002; Morgan et al., 2016) show that outward gravitational flow due to collapse of a transient central peak structure is the primary source of material in basin-scale impact craters at the distances at which we observe megabreccia (Figure 19). In both scenarios, gravitational flows are the primary depositional mechanism of megabreccia blocks in the area between the inner and outer rings of the impact basin, which is supported by the observed attributes of megabreccia in this study. However, what differs are the stratigraphic levels from which the materials participating in the gravitational flows are derived.

In hydrocode-based models described above (Collins et al., 2002; Johnson et al., 2016; Morgan et al., 2016), we may expect deep crustal/mantle materials to be present within megabreccia, including from the maximum depth of excavation (Figure 19). The smaller Orientale basin (~860-km diameter) is modeled to retain materials from 55-km depth (mantle depth) in the collapse flow (Johnson et al., 2016), while the much smaller Chixulub crater (~200-km diameter) is modeled to retain materials from 10-km depth (midcrustal depth) in the collapse flow (Morgan et al., 2016). Similar models of Isidis basin suggest that materials of >30-km depth (mantle depth) could be retained within the collapse flow (Trowbridge et al., 2019). In contrast, megabreccia formed through faulting and landslides from massive rock slope failure along the transient crater walls would represent primarily shallower materials (Figure 19). Hence, understanding the source depth of megabreccia materials will test between these two proposed models for impact basin formation. A number of orbital detections of impact megabreccia associated with lunar impact basins have revealed compositions similar to deep crustal or mantle materials containing predominantly Mg-rich LCP and olivine in a few cases (Bretzfelder et al., 2019; Klima et al., 2011; Melosh et al., 2017; Pieters et al., 1997; Yamamoto et al., 2010), suggesting that basin-scale impacts may excavate deep crust/mantle materials.

From the four lithologies determined in this study, blue blocks have LCP spectral signatures with Fe^2+^-related absorptions (Figures 2 and 6). Although the high-resolution spectral signatures of purple blocks are unknown, their HiRISE color profiles have low spectral angles and IR/BG (Figure 6) that are usually related to Fe^2+^ crystal field splitting absorptions that predominantly occur in mafic minerals (Figures 2 and 6). Based on the spectral signatures, purple and blue blocks are candidates for recording igneous materials. Furthermore, materials similar to purple blocks are not present within the other Noachian Basement Group unit. Hence, blue and purple megabreccia provide intriguing targets for the Mars 2020 rover instrument suite that could confirm/disprove the potential presence of deeply sourced materials within such igneous rocks, which we expect from models, meteorites, and orbital observations.

#### 4.3.4. Impact Melt and Ejecta

An impact basin as large as the Isidis basin is likely to have produced vast amounts of melt, excavated materials, and ejecta. However, no units within the study area clearly record such processes. In our study, we find three units (Blue Fractured Unit, Mixed Lithology Plains Unit, and LCP-bearing Plateaus Unit) that could potentially represent impact melt bearing materials.

Current understanding of the extent of impact melt sheets is primarily based on well-exposed lunar basins and Chixulub. Most impact models and empirical observations of lunar basins and Chixulub concur that the thickest melt sheet is retained within the central depression of the impact basin (inside the inner ring) (Cintala & Grieve, 1998; Hurwitz & Kring, 2014; Morgan et al., 2016; Potter et al., 2012; Spudis et al., 2014; Vaughan et al., 2013). Hence, it appears that the primary impact melt sheet produced by the Isidis basin is not exposed within the Noachian Basement Group, as all of these units extend from the inner ring to and possibly beyond the outer ring of the structure (Figures 1 and 19). Although by analogy with the lunar basin Orientale, melt deposits have been proposed around the Nili Fossae (Mustard et al., 2007). Future work may consider if any geological unit closer to the inner ring (Figure 1) is a candidate for the Isidis melt sheet.

However, melt related to the Isidis impact may be present in the form of excavated material, melt-rich ground-hugging flows (Osinski et al., 2011), smaller melt pools, and/or veneer associated with for example terracing (Cintala & Grieve, 1998). All of these units are expected to be thinner but reach larger radial distances than the central melt sheet. The Mixed Lithology Plains Unit is a candidate unit to represent a mixture of excavated, brecciated, and ejected material that is likely to contain components of melt. The unit appears geomorphologically and spectrally heterogeneous, including fractures, entrained blocks, and zones of clay formation. In addition, terminal parts of the Mixed Lithology Plains Unit appear lobate in certain cases (Figure 12). This may also explain why megabreccia and parts of Blue Fractured Unit (potentially excavated target rock) appear to occur wihin a matrix of Mixed Lithology Plains Unit.

The LCP-bearing Plateaus Unit appears to have a more limited spatial extent than the Mixed Lithology Plains Unit. Impact melt processes such as melt pool formation associated with terracing and/or other melt trapping mechanisms (e.g., topographic depressions) could be responsible for smaller concentrations of melt-rich materials. Succeeding formation of these pools, the inversion of topography would presumably be caused by differential erosion. Another possibility is limited melt-rich ejecta flow forming plateaus. Analysis of Noachian Basement outside the Isidis impact structure would aid in understanding whether LCP-bearing Plataues Unit are indeed related to the Isidis impact or have formed through a separate volcanic and/or sedimentary process. Future modeling efforts determining the extent and thickness of deposits related to the impact melt, excavation, ejecta, and gravitational flow processes during the Isidis impact would greatly improve our understanding of the units defined within the Noachian Basement Group and further our understanding of basin-scale impacts on Mars in general. Furthermore, examining these units in situ with the Mars 2020 rover would likely definitively determine their emplacement mechanism and whether they are melt/ejecta rocks.

#### 4.3.5. Several Episodes of Megabreccia Formation?

The contact between megabreccia and the Stratified Basement Unit in certain, anomalous outcrops is enigmatic, as we observe that megabreccia blocks appear to both overlie SBU within MLPU but also underlie SBU over sections of several kilometers (Figure 15). The SBU is unlikely to have formed concurrently with megabreccia as several boulderless layers (6–20) with albedo contrast and extent to outer ring are not consistent with formation through impact melt sheet, layered ejecta, or mass wasting processes. It is more likely that the SBU represents a faulted (Figure 9b) but relatively intact piece of the pre-Isidis crust. If the contact between SBU and underlying megabreccia is stratigraphic, this implies that megabreccia in the study area could potentially have two different ages (syn-Isidis and pre-Isidis). However, the contact could also be an erosional construct, allowing SBU to appear topographically above megabreccia while stratigraphically underlying megabreccia. Neither an erosional contact nor a stratigraphic boundary between SBU and underlying megabreccia can be excluded based on calculated orientations due to uncertainty associated with measurements. Determining the nature of this contact would benefit from acquiring additional stereo HiRISE images of the Western scarp of Nili Fossae where this contact may be exposed.

### 4.4. History of Hydrated Minerals and Aqueous Processes

Local abrupt color, albedo, spectral, and texture changes between Fe/Mg-smectite-bearing lithologies in yellow megabreccia and immediately surrounding Mixed Lithology Plains Unit indicate that they formed separately (Figures 2, 4, 10f, and 15d). If Fe/Mg-smectite within megabreccia and MLPU formed in a single event, color and spectral characteristics would be expected to be the same. Therefore, Fe/Mg-smectite within megabreccia most likely represent older aqueously altered target rock, while Fe/Mg-smectite within MLPU represent younger materials. Fe/Mg-smectite-bearing units lower in the stratigraphy (Stratified Basement Unit and possibly Fe/Mg-smectite Mounds Unit) differ in character compared to MLPU and predate MLPU. They potentially represent regions of intact pre-Isidis Fe/Mg-smectite-bearing Noachian Basement. These materials could be retained within target rock recorded in megabreccia, although this relationship cannot be determined from orbit with certainty. Ridges are young features that crosscut and thus formed after the formation of the SmMU, BFU, megabreccia blocks, and MLPU. Pascuzzo et al. (2019) found that all ridges contain Mg-smectite and/or mixed talc-saponite clay compositions and proposed that ridges most likely formed through shallow clastic intrusions or mineralization in fluid flows of subsurface fractures. Last, KBM in/on the MLPU are compositionally distinct and younger than most Fe/Mg-smectite-bearing materials (see section 3.3).

From these observations, we propose that the Noachian Basement Group records at least four events of hydrated mineral formation: (1) pre-Isidis Fe/Mg-smectite formation in target rock (possibly SBU and SmMU) that are now megabreccia blocks; (2) at least one and possibly several episodes of Fe/Mg-smectite formation within potential syn-Isidis impact deposits (MLPU); (3) contemporaneous or subsequent Fe/Mg-smectite formation in crosscutting fractures that now form ridges; and (4) kaolinite formation. Other questions regarding the history of hydrated mineral formation within the Noachian Basement Group are not resolvable from orbit because the mineral assemblages and rock textures are not known. Key questions include (1) Does the nature of the aqueous processes recorded each unit differ? (2) For each unit, were hydrated minerals formed as the result of a primary aqueous depositional environment or the result of diagenetic or hydrothermal processes? (3) What was the timing and nature of fluids leading to mineralization now exposed in ridges? (4) What was the timing and nature of the spatially restricted kaolinite-forming events? The various hydrated mineral-bearing lithologies provide intriguing targets for analysis and sampling with the Mars 2020 mission in order to deconvolve the complex aqueous history within the Noachian Basement Group.

### 4.5. Geological History of the Noachian Basement: Preferred Interpretation

The eight geological units and features of the Noachian Basement Group undoubtedly record a very long history of impact, igneous, and aqueous processes that happened over different geological time intervals from the Pre-Noachian or Early Noachian to Mid-Noachian (Figure 17B). Stratified Basement Unit and potentially Fe/Mg-smectite Mounds Unit represent relatively intact but deformed pieces of pre-Isidis crust. Due to their Fe/Mg-smectite compositions (Figure 6), Stratified Basement Unit, Fe/Mg-smectite Mounds Unit, and pre-Isidis target rock recorded within yellow megabreccia blocks either formed in or were affected by an aqueous environment before the formation of Isidis-related megabreccia and Mixed Lithology Plains Unit. Likewise, the Blue Fractured Unit was a target rock that predates the Isidis impact and was highly affected by the Isidis impact causing brecciation and excavation of the unit resulting in the patchy and blocky nature of the unit.

Following formation of these pre-Isidis units, megabreccia and the Mixed Lithology Plains Unit likely formed in association with the Isidis impact (Figure 17B). Megabreccia most likely represent gravitational flows associated with transient crater collapse. Some of Blue Fractured Unit and pre-Isidis Fe/Mg-smectite-bearing target rocks are recorded in blue and yellow megabreccia lithologies along with two unknown lithological components (beige and purple) that are pre-Isidis. Entrainment of excavated target rock and megabreccia materials, heterogeneity, and the occasional lobate morphological expression of the Mixed Lithology Plains Unit suggest that this unit may record a mixture of expected impact processes such as excavation, ejecta, and melt flows. These materials must have subsequently interacted with fluids causing a larger portion of these materials to become Fe/Mg-smectite-bearing (Figure 17B). The LCP-bearing Plateaus Unit postdates megabreccia and the Mixed Lithology Plains Unit, forming a younger unit of different spectral signature than Blue Fractured Unit and MLPU that did not have contact with fluids. Preferred candidate processes for the LCP-bearing Plateaus Unit formation include later melt pools or flows associated with the Isidis impact, although additional study is needed to confirm this and other igneous/sedimentary processes cannot be excluded.

The formation of ridges follows the formation of the Stratified Basement Unit, Blue Fractured Unit, megabreccia, and Mixed Lithology Plains Unit, postdating the Isidis impact through shallow clastic intrusions or mineralization in fluid flows of subsurface fractures, affecting all units stratigraphically below the LCP-bearing Plateaus Unit (Figure 17B). Likewise, kaolinite-bearing bright materials postdate the Isidis impact and formed in a separate, younger aqueous environment compared to pre-Isidis and syn-Isidis units (Figure 17B). There is no direct contact between ridges and kaolinite-bearing bright materials, but their respective lithologies suggest that they formed in separate aqueous environments.

### 4.6. Implications for Mars 2020 Rover

**Figure 20.**
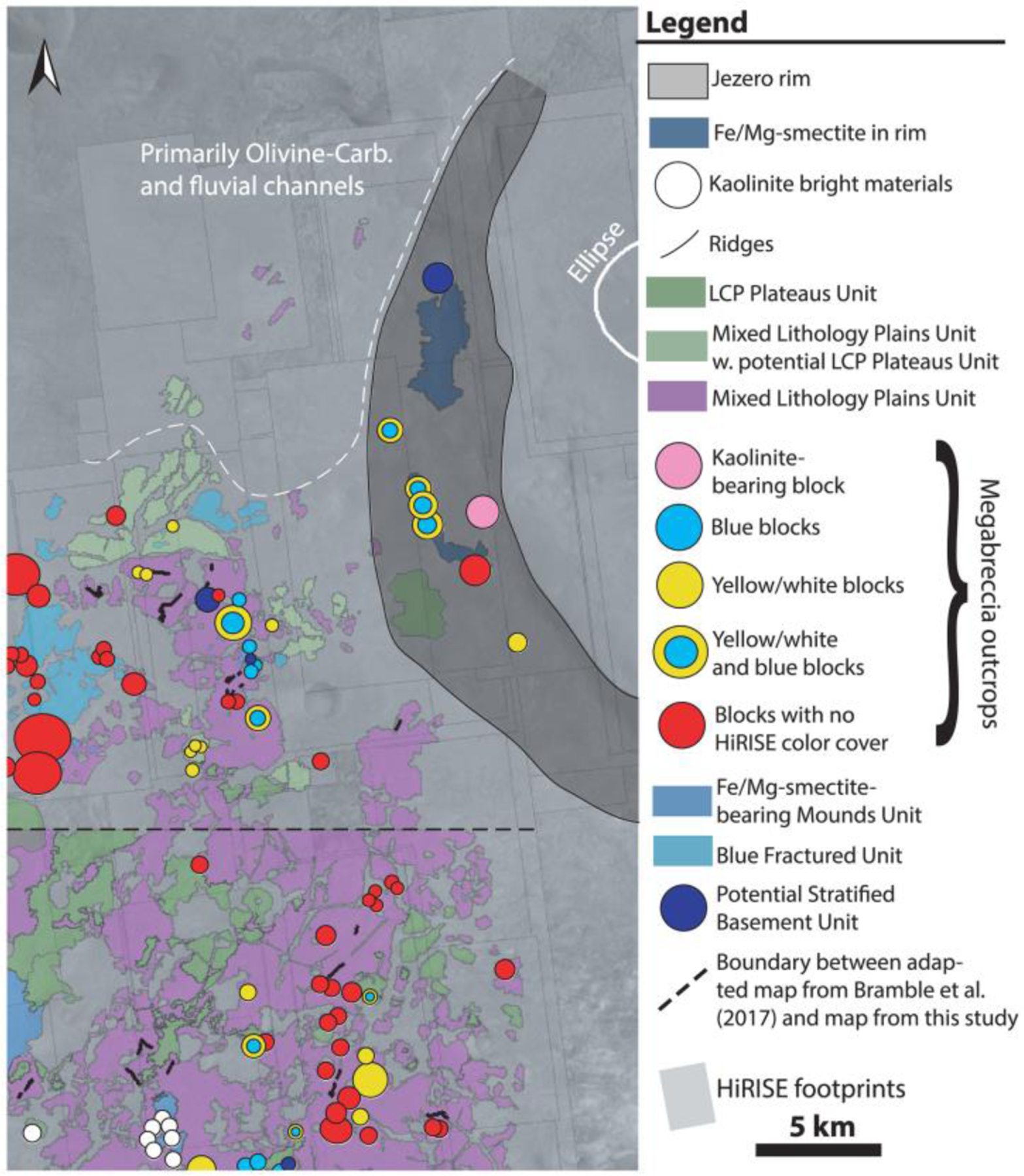
Partial map of Noachian Basement Group units and features within an area accessible by the Mars 2020 extend mission. Map of NE Syrtis (separated by black stippled line) is from Bramble et al. (2017) but adapted and modified to fit the terminology presented in this study. Megabreccia mapping scheme is based on visual characterization of HiRISE color images (see Figure 7). Note that the Jezero rim (gray shaded area) may contain areas similar to Noachian Basement Group such as LCP-bearing Plateaus Unit and Fe/Mg-smectite-bearing parts of the rim, but these are more complicated and presumably disrupted by the Jezero impact. Mixed Lithology Plains Unit with features of geomorphological similarity to LCP-bearing Plateaus Unit but no high-resolution CRISM coverage are mapped separately in light green.

The Mars 2020 instrument suite is likely to encounter eroded sediments or cobbles of Noachian Basement Group units within the walls and/or sedimentary materials within Jezero crater because the Jezero watershed includes large areas of Noachian Basement Group units (Goudge et al., 2015). In addition, the entire Noachian Basement Group may be explored in situ with a ~20-km extended mission that would take the Mars 2020 rover west of the Jezero rim (Figure 20), answering many outstanding questions both about the regional geology and about ancient terrestrial planetary processes in general (Table 4).

First, identifying excavated mantle materials within megabreccia would have implications not only for Mars mantle petrology and understanding the composition of the Martian mantle and resulting melting pathways but also for our understanding of basin-scale impact models. The presence or nonpresence of mantle materials within megabreccia would provide an important depth constraint to excavation for basin-scale impact models. Blue, yellow, and purple megabreccia lithologies are present in an extended mission traverse (Figure 20). Megabreccia, including one kaolinite block, are even present in the rim of Jezero crater although it cannot be discerned whether these were formed through the Jezero crater or the Isidis basin. In addition, it is highly likely that the Noachian Basement Group records impact melt from the Isidis basin in one of its three LCP-bearing units.

The Mars 2020 rover would also be able to analyze the Mixed Lithology Plains Unit, LCP-bearing Plateaus Unit, megabreccia, and Blue Fractured Unit that may provide examples of geological units with different igneous origins or at least different aqueous alteration processes. The importance of understanding the igneous compositions of the Noachian Basement is furthered by the presence of HCP higher in the regional stratigraphy, often referred to as the mafic cap unit in literature (Figure 1) (Bramble et al., 2017; Ehlmann et al., 2009; Goudge et al., 2015; Mustard et al., 2009). From orbital data and petrological modeling efforts, it has been suggested that there was a global transition from LCP-dominated to HCP-dominated igneous compositions on the Martian surface related to thermal evolution of the Martian mantle (Mustard et al., 2005; Baratoux et al., 2013). The three different LCP-bearing Noachian Basement units may potentially also record changes in composition from lithologies containing more Fe-rich LCP compositions to lithologies containing more Ca-rich LCP compositions. However, due to the uncertainties regarding the spectral signatures of these materials discussed above, this cannot be confirmed from orbit. However, this can be confirmed by analysis with instruments on the Mars 2020 rover. Therefore, analyzing and understanding the Noachian basement LCP-bearing units, their relationship to each other, and their transition to the younger HCP-bearing units may have implications for understanding the Martian mantle evolution, its melting processes, and surface volcanism on Mars.

Lastly, the Mars 2020 rover will be able to provide detailed analyses of petrographic texture, composition, mineral assemblages, and stratigraphy and thus infer habitability and environmental transitions recorded by the several units in the Noachian Basement Group with hydrous materials, including Stratified Basement Unit, Mixed Lithology Plains Unit, Fe/Mg-smectite-bearing megabreccia, ridges, and KBM. First, the origin of layering in smectite-bearing pre-Isidis Stratified Basement Unit could be sedimentary or volcanic. Second, the Mixed Lithology Plains Unit appears to represent a spatially extensive aqueous environment, and it still remains to be answered why hydrated mineralogy formed on such a large scale on Noachian Mars and whether this was impact related. The vast Fe/Mg-smectite formation on Mars has had many explanations proposed in previous literature, including subsurface alteration, hydrothermalism, burial metamorphism/diagenesis, and pedogenetic processes (Ehlmann & Mustard, 2012; Ehlmann et al., 2011; Mustard et al., 2009; Viviano et al., 2013). Detailed mineralogical, chemical, and textural studies of these units will reveal the temperature and fluid chemistry of formation, testing between these different aqueous environments. The megabreccia may preserve very ancient pre-Isidis water-related processes. The ridges, in contrast, represent a separate syn- or post-Isidis episode of fluid flow, possibly unrelated to the original Fe/Mg-smectite. Last, the relationship between Fe/Mg-smectite clays and overlying KBM observed in the study area may reveal hints to similar relationships observed globally when studied in situ (Carter et al., 2014; Ehlmann et al., 2011). Hence, the many units with hydrous minerals in the Noachian Basement Group record multiple different aqueous environments on ancient Mars that will be revealed through in situ analysis and sample return.

## 5. Conclusions

We define the oldest, lowermost stratigraphy west of the Isidis basin to be a Noachian Basement Group composed of five distinct geological units (Stratified Basement Unit, Blue Fractured Unit, Mixed Lithology Plains Unit, LCP-bearing Plateaus Unit), two geomorphological features (megabreccia and ridges), and one mineral deposit (kaolinite-bearing bright materials). The stratigraphically lowermost units are the Stratified Basement Unit, Blue Fractured Unit, and pre-Isidis megabreccia materials. The Stratified Basement Unit contains Fe/Mg-smectite-bearing materials layered (6–20 layers/exposure) at scales of tens of meters or less. The Stratified Basement Unit contains polygonally fractured terrain containing low-Ca pyroxene with strong Fe^2+^ absorptions and little to no alteration. The overlying Mixed Lithology Plains Unit is in the middle of the stratigraphy and contains vast, usually smooth plains of LCP and Fe/Mg-smectite mixed together with diffuse boundaries between LCP-dominated and Fe/Mg-smectite-dominated parts of the plains. The Mixed Lithology Plains Unit also sometimes contains megabreccia and patches of Blue Fractured Unit. Stratigraphically above the Mixed Lithology Plains Unit is the LCP-bearing Plateaus Unit: flat, raised plateaus that contain large areas of completely unaltered LCP. Fe/Mg-smecite-bearing ridges and kaolinite-bearing bright materials occur within the Mixed Lithology Plains Unit and likely represent the youngest features in the Basement Group.

The megabreccia are observed primarily within the NW part of the Isidis basin structure 500–1,000 km from the crater center within the Mixed Lithology Plains Unit. They are angular to subrounded, have diverse block packing density, and are sometimes layered, and block lithology is often heterogeneous within a single outcrop. Through parameterization of HiRISE color images, we find four different lithologies indicated by yellow/white, blue, beige, and purple colors in HiRISE false color. CRISM data show yellow/white and blue materials contain Fe/Mg-smectite and Blue Fractured Unit type LCP, respectively. However, beige and purple megabreccia do not occur at a sufficient spatial scale in any areas with CRISM coverage to examine their composition and are likely distinctive pre-Isidis materials (Pre-Noachian or Early Noachian). Block sizes of megabreccia ranged from 1.3–433 m with a median of 11.5 m with no clear correlation to distance from crater center or elevation. Taken together, the heterogeneity, sedimentological properties, block size, and spatial extent/distribution of megabreccia appear to be most compatible with formation through gravitational flows resulting from collapse of the transient crater during Isidis basin formation.

The LCP-bearing Plateaus Unit, Mixed Lithology Plains Unit, and Blue Fractured Unit all contain LCP but differ in spectral characteristics related to either pyroxene composition (high vs. low Ca) and/or glass content. This suggests that at least three different types of LCP-bearing lithologies have formed at different stratigraphic times. These have also undergone different degrees of aqueous alteration within the Noachian Basement Group. Similarly, four aqueous alteration events of (1) pre-Isidis Fe/Mg-smectite formation, (2) Fe/Mg-smectite formation within potential impact deposits, (3) Fe/Mg-smectite formation in fractures forming ridges, and (4) kaolinite formation are responsible for formation of hydrated mineralogy within the Stratified Basement Unit, Fe/Mg-smectite-bearing Mounds Unit, Mixed Lithology Plains Unit, ridges, and kaolinite-bearing bright materials.

Outstanding questions include four major topics. (1) What was the duration and evolution of aqueous processes giving rise to hydrated mineralogy of different stratigraphic ages stretching from the Pre-Noachian or Early Noachian to Mid-Noachian? (2) What igneous or impact process(es) formed LCP, potentially OPX, within the pre-Isidis crust? (3) Do megabreccia contain mantle materials and what does that tell us about the Martian interior? (4) What processes of 1,900-km Isidis impact basin formation are recorded within the Noachian Basement Group and does this match predictions of current basin formation models? Many of these questions are answerable with in situ exploration and sampling by the Mars 2020 mission in an extended mission taking the rover ~20 km from the Jezero landing ellipse.

## Supplementary Material

Suplpement 1

Supplement 3

Supplement 4

Supplement 2

## Figures and Tables

**Table 1:** Expected Characteristics of Megabreccia Deposits From Four Known Megabreccia Formation Mechanisms

Formation mechanism	Distribution	Texture/lithology	Block sizes	References
Ballistic ejecta	Circumferential to outer and inner crater ring Extend >2 crater radii	Multitude of textures/lithologies Potential sorting of textures/lithologies with distance	Potential dependency on distance from crater center	Hörz (1982)
Ground-hugging, ejecta-related melt flows	Not necessarily circumferential Should extend beyond the outer ring Occurs locally	Significant melt component Flow/dike/pseudotachylite structures	No particular expectations	Komatsu et al. (2007) and Osinski et al. (2011)
Crater floor/central peak fracturing and/or melt sheet formation	Occurs within inner ring or central peak	Uplifted/faulted blocks Preimpact lithologies Should be in matrix of melt	Primarily large blocks (100s of meters)	Caudill et al. (2012), Schultz (1976), Quantin et al. (2012), and Krüger et al. (2016)
Gravitational flow during crater collapse	Circumferential to inner crater rim Occurs primarily within transient crater and faulted region (likely between outer and inner ring)	Multitude of textures/lithologies Evidence for ground transport No spatial sorting, complete heterogeneity	Potential dependency on elevation but not distance	Belza et al. (2012), Stöffler et al. (2004), and Trowbridge et al. (2019)

**Table 2:** Data Sets With Their Related Online Repositories and References Used in This Study

Data set name	Online repository	Reference
HiRISE RDR	Planetary Data System (PDS)	McEwen et al. (2007)
CRISM TRR3, MSP, and MSW	Planetary Data System (PDS)	Murchie et al. (2007)
MOLA global mosaic	Planetary Data System (PDS)	Zuber et al. (1992)
CTX global mosaic	Murray Lab/ArcGIS online	Dickson et al. (2018)
THEMIS daytime/nighttime global mosaic	ASU Mars Global Data Sets	Edwards et al. (2011)

**Table 3:** Summary Table of Geological Units (Plain Text), Geomorphological Features (in Italics), and Mineral Deposit (in Italics and Bold) Within the Noachian Basement

Unit/feature name	Acronym	CRISM composition	Geomorphology	Stratigraphic level/time order	Relevant figure(s)
Stratified Basement Unit	SBU	Fe/Mg-smectite	Individual layers have a thickness of 8–42 m Between 6 and 20 layers in each exposure Exposures have a horizontal extent of 300 m to 10 km	Lower	Figures 8–9
Blue Fractured Unit	BFU	LCP (low centroid) Occasional, minor Fe/Mg-smectite	Highly fractured polygonal patches of bedrock Distinct blue color in HiRISE	Lower	Figures 8–10
Fe/Mg-smectite Mounds Unit	SmMU	Fe/Mg-smectite	Topographic highs with sharp contact to the Mixed Lithology Plains Unit Ridged and knobby mounds	Unknown-potentially lower	Figures 8 and 12
*Megabreccia*	*MB*	*LCP (low centroid) Fe/Mg-smectite Unknown for beige- and purple-colored blocks in HiRISE*	*Angular or subrounded blocks with abrupt textural contrast to surrounding matrix materials* *Distinct blue, yellow, beige, and purple colors in HiRISE*	*Lower-middle depending on outcrop relationship with SBU and MLPU*	Figures 8, 14, and 15
Mixed Lithology Plains Unit	MLPU	Mixture of LCP (high centroid) and Fe/Mg-smectite	Low-lying plains Generally heterogeneous with fractured, knobby, or smooth terrains	Middle	Figures 8 and 10–12
LCP-bearing Plateaus Unit	LPU	LCP (middle centroid); no evidence of alteration	Topographic highs Smooth, flat plateaus	Upper	Figures 8 and 10–12
*Ridges*	*R*	*Fe/Mg-smectite*	*Semilinear ridges May occur in six different geometric configurations* (Pascuzzo et al., 2019 *Bright white or yellow in HiRISE*	*Younger-no known contact to LPU, SmMU, and KBM*	Figures 8 and 16
*Kaolinite-bearing bright materials*	***KBM***	***Kaolinite***	***Irregular, bright, white patches of hundreds of meters***	***Younger-younger than LPU***	Figures 8 ***and*** 13

**Table 4:** Summary Table of Geological Units and Related Science Questions Within an Extended Mission From Jezero Crater

Unit name	Distance to Jezero ellipse	Science objectives/questions at units
Kaolinite-bearing bright materials	~9 km	Detailed mineralogical and chemical analysis of kaolinite-bearing bright materials will reveal how the related aqueous environment(s) differed or were similar to the environment(s) that formed Fe/Mg-smectite.
Ridges	~22 km	Detailed mineralogical and chemical analysis of materials within ridges may reveal the chemistry and temperature of fluid flow within ridges. Understanding the chemistry of fluids may help us understand the habitability potential of these fracture systems.
LCP-bearing Plateaus Unit	~13 km	Analyzing the texture of LCP-bearing Plateaus Unit with Mars 2020 cameras may reveal whether the LCP-bearing Plateaus Unit have an impact, sedimentary, and/or igneous origin. Detailed mineralogical and chemical study will reveal what type of environment the LCP-bearing Plateaus Unit formed in (e.g., lava flow, impact melt flow, and lithified sandstone).
Mixed Lithology Plains Unit	~9 km	Analyzing the texture of the Mixed Lithology Plains Unit with the Mars 2020 cameras will reveal whether the Mixed Lithology Plains Unit is an impact product or formed through different processes. Detailed mineralogical and chemical study of the hydrated materials within the Mixed Lithology Plains Unit with the Mars 2020 instrument suite may reveal the aqueous environment(s) that formed them. Analysis of these aqueous environment(s) will help us understand ancient Mars habitability and climate.
Fe/Mg-smectite in the Jezero rim	~6 km	Detailed mineralogical and chemical study of the hydrated materials within the Fe/Mg-smectite Mounds Unit with the Mars 2020 instrument suite may reveal the aqueous environment(s) that formed them. Analysis of these aqueous environment(s) will help us understand ancient Mars habitability and climate.
Megabreccia	~9 km	Detailed mineralogical and chemical study will reveal the composition of the earliest Martian crust that are captured within megabreccia materials. Determining the source depth of megabreccia materials will enable us to test between impact basin models.
Blue Fractured Unit	~18 km	Detailed mineralogical and chemical study with the Mars 2020 instrument suite can easily determine whether the Blue Fractured Unit is deep crustal/mantle materials or other igneous materials. Sampling and in situ analysis of the Blue Fractured Unit will potentially inform us on the deep crustal/mantle composition of Mars and/or other igneous petrogenetic processes.
Potential Stratified Basement Unit	~7 km	Analyzing sedimentary or volcanic layers in the Stratified Basement Unit will inform interpretations of the depositional environments and processes by which layers and extensive Fe/Mg-smectite clays formed within the Stratified Basement Unit. Analysis of these aqueous environment(s) will help us understand ancient Mars habitability and climate.

## Key Points:

- The Noachian Basement Group contains a newly defined stratigraphy of five geological units and three geomorphological or mineralogical features
- Isidis-related megabreccia likely formed through mass wasting during transient crater collapse and record four distinct pre-Isidis lithologies
- A M2020 extended mission would provide deep crust, impact melt, and hydrated lithologies of distinct Pre-Noachian or Early Noachian ages
