# Supplementary material for: Composition, Stratigraphy, and Geological History of the Noachian Basement Surrounding the Isidis Impact Basin
Source: J Geophys Res Planets. Author manuscript; Available in PMC 2021 Aug 20. (PMC8378244; doi:10.1029/2019je006190)
Supplement: Supplement 4 [file NIHMS1730637-supplement-Supplement_4.docx]

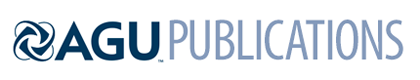


*Journal of Geophysical Research*

Supporting Information for

**Composition, Stratigraphy, and Geological History of the Noachian Basement Surrounding the Isidis Impact Basin**

Scheller E. L.^1^ and Bethany, B. L.^1,2^

^1^Division of Geological and Planetary Sciences, California Institute of Technology, Pasadena, California, USA. ^2^Jet Propulsion Laboratory, Pasadena, California Institute of Technology, Pasadena, California, USA.

**Contents of this file**

Captions for dataset S1-S3

**Additional Supporting Information (Files uploaded separately)**

Dataset S1
Dataset S2
Dataset S3

**Introduction**

The supporting information includes 3 tables (uploaded separately) in the form of tab-delimited txt-files. MOLA longitudinal and latitudinal coordinates, MOLA elevation, and distance of outcrop to Isidis crater center in all 3 datasets were extracted in ArcGIS.

- Supplement 1 includes a table of all megabreccia outcrops mapped in this study. The table includes relevant MOLA coordinates (longitude, latitude, and elevation), texture, and HiRISE color information for each megabreccia outcrops. Texture and HiRISE color categories were assigned visually according to categories determined in the study. For some megabreccia outcrops, HiRISE color information was not available and these fields were left blank.
- Supplement 2 includes a table of all individual blocks of megabreccia clasts that were analyzed for size. Block sizes were calculated as the diameter of the minimum enveloping circle to each block outline in ArcGIS.
- Supplement 3 includes a table of line segments from outcrops of Stratified Basement Unit from 2 different HiRISE DEMs (ESP_016153_2005 and ESP_019476_2005) and their calculated orientations (strike, dip, rake error min, and rake error max). Orientations of each line segment was calculated using the Attitude python package (Quinn and Ehlmann, 2019 b). A few of these line segments yield orientations that are anomalous and have large associated rake error ($\theta_{max}$>9º).

Data Set Supplement 1: Outcrop Texture and Color. This data set includes all mapped megabreccia outcrops in the study. Positions for each megabreccia outcrop are given as MOLA longitudinal and latitudinal coordinates in the SimpleCylindrical_Mars projection extracted through ArcGIS, MOLA elevation, and distance of outcrop to Isidis crater center. It also includes the texture and HiRISE color properties of each megabreccia outcrop as determined in this study.

Data Set Supplement 2: Block sizes. This data set includes all blocks of megabreccia clasts analyzed for size properties. Positions for each block of megabreccia clasts are given as MOLA longitudinal and latitudinal coordinates in the SimpleCylindrical_Mars projection extracted through ArcGIS, MOLA elevation, and distance of outcrop to Isidis crater center.

Data Set Supplement 3: Stratified Basement Orientations. This data set includes all orientations calculated from line segments of Stratified Basement Unit using the Attitude python package (Quinn and Ehlmann, 2019b). The table includes strike, dip, and rake error results.
